# Evaluation of a Web-Based Intervention to Promote Hand Hygiene: Exploratory Randomized Controlled Trial

**DOI:** 10.2196/jmir.1963

**Published:** 2011-12-09

**Authors:** Lucy Yardley, Sascha Miller, Wolff Schlotz, Paul Little

**Affiliations:** ^1^Academic Unit of PsychologyFaculty of Human and Social SciencesUniversity of SouthamptonSouthamptonUnited Kingdom; ^2^Institute of Experimental PsychologyUniversity of RegensburgRegensburgGermany; ^3^Primary Care and Population SciencesFaculty of MedicineUniversity of SouthamptonSouthamptonUnited Kingdom

**Keywords:** Health promotion, human influenza, hand-washing, randomized controlled trial

## Abstract

**Background:**

Hand-washing is regarded as a potentially important behavior for preventing transmission of respiratory infection, particularly during a pandemic.

**Objective:**

The objective of our study was to evaluate whether a Web-based intervention can encourage more frequent hand-washing in the home, and to examine potential mediators and moderators of outcomes, as a necessary first step before testing effects of the intervention on infection rates in the PRIMIT trial (PRimary care trial of a website based Infection control intervention to Modify Influenza-like illness and respiratory infection Transmission).

**Methods:**

In a parallel-group pragmatic exploratory trial design, 517 nonblinded adults recruited through primary care were automatically randomly assigned to a fully automated intervention comprising 4 sessions of tailored motivational messages and self-regulation support (n = 324) or to a no-intervention control group (n = 179; ratio 2:1). Hand-washing frequency and theory of planned behavior cognitions relating to hand-washing were assessed by online questionnaires at baseline (in only half of the control participants, to permit evaluation of effects of baseline assessment on effect sizes), at 4 weeks (postintervention; all participants), and at 12 weeks.

**Results:**

Hand-washing rates in the intervention group were higher at 4 weeks than in the control group (mean 4.40, n = 285 and mean 4.04, n = 157, respectively; *P* < .001, Cohen *d* = 0.42) and remained higher at 12 weeks (mean 4.45, n = 282 and mean 4.12, n = 154, respectively; *P* < .001, Cohen *d* = 0.34). Hand-washing intentions and positive attitudes toward hand-washing increased more from baseline to 4 weeks in the intervention group than in the control group. Mediation analyses revealed positive indirect effects of the intervention on change in hand-washing via intentions (coefficient = .15, 95% confidence interval [CI], .08–.26) and attitudes (coefficient = 0.16, 95% CI, .09–.26). Moderator analyses confirmed that the intervention was similarly effective for men and women, those of higher and lower socioeconomic status, and those with higher and lower levels of perceived risk.

**Conclusions:**

This study provides promising evidence that Web-based interventions could potentially provide an effective method of promoting hand hygiene in the home. Data were collected during the 2010 influenza pandemic, when participants in both groups had already been exposed to extensive publicity about the need for hand hygiene, suggesting that our intervention could add to existing public health campaigns. However, further research is required to determine the effects of the intervention on actual infection rates.

**Trial:**

International Standard Randomized Controlled Trial Number (ISRCTN): 75058295; http://www.controlled-trials.com/ISRCTN75058295 (Archived by WebCite at http://www.webcitation.org/62KSbkNmm)

## Introduction

Respiratory infections, such as influenza and even the common cold, continue to present a major health problem in the 21st century. Influenza pandemics have the potential to cause substantial morbidity and mortality as well as widespread social and economic disruption [1]. While the 2009 H1N1 pandemic proved relatively mild for most people, a much more severe influenza pandemic (eg, H5N1) is still anticipated, which could result in many millions of deaths worldwide [2]. In nonpandemic years, colds and influenza still pose a considerable burden for individuals, health services, and society through their impact on quality of life, the ability to work, vulnerability to more serious illness, and need for medical care [3,4].

The relative importance of different routes of infection by influenza has not yet been established, but the current consensus is that transmission from hand to face could play a significant role [5]. Adoption of simple preventive hygiene measures, especially frequent hand-washing, could prove a cost-effective means of reducing transmission of respiratory infections [6-9], and these measures were therefore recommended during the H1N1 pandemic by the World Health Organization and promoted in national campaigns worldwide. Slowing the spread of infection could help to prevent health and other services from becoming overwhelmed and allow time for the development and distribution of vaccines [10]. However, surveys carried out in the context of both severe acute respiratory syndrome (SARS) and influenza pandemics have found that less than half of those surveyed reported adhering to recommended rates of hand-washing (at least 10 times a day), in both community and higher-risk samples [11-14]. Adherence to hygiene recommendations is probably lower than these surveys suggest, since self-reported hand-washing rates typically overestimate actual hand-washing behavior [15].

There is clearly a need to develop interventions to promote hygienic behavior and test their effectiveness. Interventions are required that could be made available to the general public rapidly and at low cost, since most of the population is likely to be at risk from pandemic influenza [1]. The Internet seems an ideal medium for such an intervention; in a survey carried out in the United States, most respondents stated that the Internet would be the first source of information that they would consult in the event of a pandemic [16]. However, we are aware of only one small study of a Web-based intervention to reduce transmission of influenza [17], which found positive trends in behavior but no significant effect on hand hygiene.

When developing public health interventions, whether online or offline, it is important not only to demonstrate effectiveness but also to establish what sectors of the population can be reached by each type of intervention employed, and in particular to ensure that interventions reach those most in need of them [18,19]. While the Internet may be the best medium for reaching much of the population, it may be less effective for some sectors, such as older people and socially deprived groups [20]. Previous pandemics (including the recent H1N1 pandemic) have had a more severe impact on these sectors of the population, which are typically more vulnerable to health problems [21-24]. It is therefore vital to consider whether a Web-based hygiene intervention could be used to reach older and socially deprived people, or whether alternative interventions may be required. In addition, hand-washing rates are known to be lower in men and those less concerned about risk of infection [12,14,15,25,26], and so it is necessary to evaluate whether the intervention is effective in men and those with low perceived risk.

### Developing and Testing the Intervention

Our Web-based intervention to promote hygienic behavior was developed following best practice for theory- and evidence-based intervention development [27-30]. The most appropriate target behaviors and the key attitudes and beliefs associated with these behaviors were identified by literature review and a series of qualitative and quantitative pilot studies [31,32]. The theory of planned behavior [33] was used as the principal theoretical framework, as it is flexible enough to be applied in a wide variety of contexts, it can be combined with other models and predictors, and there is evidence that components of the model that are amenable to change by intervention are key predictors of health-related behavior [34-36]. The theory of planned behavior proposes that any behavior is determined principally by the intention to perform that behavior. Intention is in turn determined by (1) attitude (a global evaluation of whether performing the behavior will have positive or negative outcomes), (2) subjective norm (the perception that relevant others would approve or disapprove of the individual carrying out the behavior), and (3) perceived behavioral control (the extent to which the individual feels it is easy or difficult to carry out the behavior). We therefore applied the model by constructing messages that would promote positive attitudes, subjective norms, and perceived behavioral control by encouraging participants to perceive hand-washing as effective, socially desirable, and easy to do. These were supplemented by theory-based techniques addressing perceived risk of pandemic flu [37], promoting appropriate illness perceptions [38], and supporting implementation of intended behavior [39,40]. In total, the intervention incorporated 18 of the 26 theory-based behavioral change techniques listed in a recently published taxonomy [41]. Our intervention was developed with input from all sectors of the community and was designed to be accessible and appropriate for men and women of all ages, of high and low socioeconomic status, and with a high and low perceived risk of infection [31].

The present study was designed to test the effects of our Web-based intervention on hand hygiene, as an essential precursor to a pragmatic trial of the effects on infection transmission. We hypothesized that hand-washing rates, and intentions to wash hands more frequently in the future, would be higher in those given access to the intervention than in those who were not given access to it. We tested this prediction at 4 weeks (immediately after completing the intervention) and at 12 weeks, to check whether any increase in hand-washing was maintained. We also tested the prediction that the theory of planned behavior cognitions targeted by the intervention (ie, intentions, attitudes, subjective norm, and perceived behavioral control) would increase more from baseline in the intervention than in the control group, and that changes in cognitions would mediate changes in behavior. To examine potential variations in response to the intervention in different sectors of the population, we then analyzed the effects on hand-washing of age, gender, socioeconomic status, and perceived risk of infection. We hypothesized that there would be no moderator effects on intervention outcome, despite any baseline differences in hand-washing rates that might be found.

## Methods

### Design

Ethics approval was obtained from the National Research Ethics Service. In a parallel-group design, when participants initially logged on to the website, two-thirds were automatically randomly assigned by the intervention software to receive the intervention and one-third to the control condition, which received no intervention. No blinding of participants was possible, nor would it have been appropriate to our pragmatic design [42].

In pragmatic trials it is considered good practice to avoid intervening in the control group in any way that might change outcomes and therefore affect the comparison of effect sizes in the intervention and control groups [42]. If measurement of attitudes and behavior might affect outcomes [43], it is necessary to omit measurement until the intervention has been delivered. The rationale is that effects of measurement on behavior are likely to be greater in the control than in the intervention group, and will therefore lead to an underestimation of the intervention effect that would be observed if the intervention were implemented. For example, asking participants to answer questions that require them to reflect on their hand-washing behavior might influence behavior in a control group with no other intervention, but may not have any additive effect on the behavior of an intervention group that is exposed to extensive materials encouraging such reflection. However, it is considered good practice in behavioral research to control for measurement effects and to examine mediators of intervention effectiveness by comparing change in attitudes and behavior from baseline in the intervention and control groups. Since this behavioral study was designed as the precursor to a pragmatic trial, we felt it was important to satisfy both these requirements. We therefore randomly assigned our control participants to two subgroups: one received all the same measures as the intervention group, while the other completed measures only at 4 weeks and 12 weeks. This solution enabled us to estimate intervention effects in the absence of any contamination of control group behavior, but also allowed us to check that intervention effects could not be attributed to mere measurement.

### Intervention

**Figure 1:**
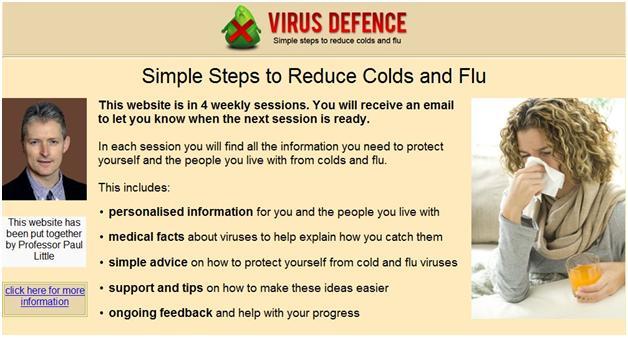
Homepage of the Internet Doctor website.

The intervention consisted of four weekly Web-based sessions, each containing new content in order to encourage repeat visits [44,45]. See Figure 1 and Multimedia Appendix 1 for illustrative screen shots, Multimedia Appendix 2 for more details of the intervention development and content, and http://www.lifeguideonline.org/player/play/primitdemo for demonstration pages from the first session (archived by WebCite at http://www.webcitation.org/634AW68U7). Session 1 (10 core pages) provided all the essential components of the intervention, including information about the medical team giving the advice (to enhance credibility); the need to prevent seasonal and pandemic flu; the link between hand-washing and virus transmission; expert recommendations for hand-washing frequency and technique; and instructions for picking up a free supply of hand gel from their local practice. Participants completed a hand-washing plan to promote intention formation with situational cueing. Tailored feedback was provided to help users improve their plan where necessary. Users were encouraged to print, sign, and post up the plan and involve other household members.

The three remaining sessions reinforced positive attitudes and norms and addressed common negative beliefs identified during piloting. Tailored feedback was given based on 3 items assessing current hand-washing frequency, agreement that hand-washing would prevent virus transmission, and perceived difficulty of carrying out the behavior. On logging on to the second session, half of the participants were randomly assigned to also receive advice (1 page per session) on how to reduce infection risk by boosting the immune system (eg, through a healthy lifestyle or taking echinacea). The purpose of this comparison was to check that risk-compensation mechanisms [46] did not lead to a reduction in hand-washing rates because advice on other methods of reducing infection had been given. Comparison of the intervention groups with and without these additional pages revealed absolutely no differences in outcomes, and so both intervention subgroups were pooled for analysis.

### Participants and Procedure

Participants were recruited by mailed invitations to take part in a study of methods of reducing the spread of infection from colds and seasonal and pandemic flu. These were sent to 8150 people aged over 18 years randomly sampled from the lists of nine general practices in Southern England from August to October 2010 (4 months after the onset of the H1N1 pandemic), including practices in areas of high and low socioeconomic deprivation. The invitation letter (Multimedia Appendix 3) sought participation from people with home Internet access and living with at least one other household member.

After returning their signed consent forms and email address, participants were emailed a unique username and URL for logging on to the website. Participants who were allocated baseline assessments completed them online on their initial login. Following first login, participants in the intervention groups were emailed after 4 days to log in to session 2, and invitations to sessions 3 and 4 followed at 1-week intervals after login to the previous session (see Table 1 for an overview of study procedures and Multimedia Appendix 4 for the protocol). To prompt usage, two follow-up emails were sent to participants who did not log in to any session [44,45].

**Table 1:** Overview of study procedures

Time point	Intervention group	Control group with baseline measurement	Control group without baseline measurement
Recruitment	Informed consent; collection of personal details; initial login; randomization	Informed consent; collection of personal details; initial login; randomization	Informed consent; collection of personal details; initial login; randomization
Baseline	Assessment of hand-washing rates, theory of planned behavior cognitions, perceived risk	Assessment of hand-washing rates, theory of planned behavior cognitions, perceived risk	No assessment
Weeks 0–3	Weekly email invitations to log on to Web-based session promoting hand-washing	No intervention	No intervention
Week 4	Assessment of hand-washing rates, theory of planned behavior cognitions	Assessment of hand-washing rates, theory of planned behavior cognitions	Assessment of hand-washing rates, theory of planned behavior cognitions
Week 12	Assessment of hand-washing rates, theory of planned behavior cognitions	Assessment of hand-washing rates, theory of planned behavior cognitions	Assessment of hand-washing rates, theory of planned behavior cognitions

All participants were sent invitations to complete the assessment measures online at 4 weeks and 12 weeks after initial login (regardless of progress through the sessions). Two follow-up emails were sent for each assessment. To maximize follow-up, phone calls were made to nonresponders to the 4- and 12-week assessments to elicit responses to the primary outcome measure (hand-washing frequency).

### Measures

Hand-washing frequency (explicitly defined as using soap and water or antibacterial gel) was assessed by a single item ranging from 1 (0–2 times a day) to 5 (10 or more times a day). Intentions were measured by a 3-item scale asking the respondent to indicate on a 7-point scale (from 1 = disagree strongly to 7 = agree strongly) that they intended to wash their hands “at least 10 times a day,” “more often,” and “as often as possible” (alpha = .91). Self-reported frequency of hand-gel use was also assessed by a single item ranging from 1 (0–2 times a week) to 5 (10 or more times a week).

All measures of theory of planned behavior cognitions and perceived risk were also scored from 1 to 7; items were recoded for analysis where necessary so that higher scores indicate greater agreement, and summed subscale scores were divided by the number of items to allow direct comparison. All items assessing theory of planned behavior cognitions explicitly elicited views of hand-washing with soap or antibacterial gel at least 10 times a day (the key target behavior for the intervention). Attitudes were measured by 6 bipolar semantic differential scales: 3 items formed a direct measure of instrumental attitude (asking whether the target behavior was seen as useless/useful, unnecessary/necessary, or bad/good), and 3 measured affective attitude (asking whether the target behavior would make the respondent feel worried/confident, proud/embarrassed, or sensible/foolish). However, factor analysis indicated that these items clearly loaded on a single scale (alpha = .92): 2 items (alpha = .90) assessed subjective norms by measuring agreement (7 = agree strongly) that “people whose opinions matter to me” and “people I live with” would approve of the target behavior. Perceived behavioral control for carrying out the target behavior was assessed by 2 items (alpha = .95) measuring the self-efficacy (“I am confident that I could”) and perceived control (“it will be possible for me”) dimensions. Respondents indicated agreement with these statements (7 = agree strongly), which were preceded by “If I wanted to,” to hold motivation constant [47,48].

Perceived risk of infection was assessed by agreement (7 = agree strongly) with 2 items (alpha = .90) assessing perceived likelihood of catching pandemic flu if no preventive action was taken [49]. This dimension of risk was assessed because pilot work indicated it was a better predictor of hand-washing intentions than was perceived worry about infection or perceived severity of infection [31,50].

Participants reported their gender, age, and postcode. The GeoConvert program [51] was used to estimate socioeconomic status from postcode, based on the Indices of Deprivation 2007 Lower Super Output Area Score (England), the official UK government measure of the relative socioeconomic deprivation associated with each postcode area, based on a weighted combination of 37 different indicators (a lower ranking denotes less deprivation). Website usage was analyzed by number of sessions accessed [52]. Practice staff kept a record of which participants collected their free sample of hand gel.

### Statistical Analysis

The effectiveness of the intervention was tested first by a direct comparison (by independent *t* test, using Cohen *d* to assess effect size) of the primary and secondary outcome measures, hand-washing frequency and intentions, in the control and intervention groups at 4 weeks and 12 weeks, based on all participants who provided data at each time point. To examine possible measurement effects on outcomes, we repeated the between-group analyses at 4 weeks, comparing the intervention group with the control groups with and without baseline measurement. This analysis was not repeated for the 12-week follow-up since by that time point both control groups had been exposed to the measures. We powered the study to have 80% power to detect a small to medium effect size (*d* = 0.35) in the key comparison between the control and intervention groups with alpha = 0.05; this required a minimum sample size of 97 in the control group and 195 in the intervention group. We chose this effect size since effect sizes of Web-based interventions are typically quite small (though nevertheless potentially useful at a population level), but very small effects were not worth detecting, as they would not be clinically useful.

We further examined intervention effects by mixed-effects regression models for longitudinal data comparing change in intentions from baseline to 4 weeks in the control and intervention groups. Mixed-effects regression models were also employed to compare change in the theory of planned behavior cognitions between baseline and 4 weeks. Mixed-effects regression models use all available data within subjects, so that there is no need to replace missing values.

To examine whether intervention effects on behavior were a consequence of changes in cognitions, we used mediation analysis to test indirect effects of intervention on change in hand-washing behavior via changes in those cognitions that were targeted to be modified by the intervention. We estimated confidence limits of the total indirect effect by bias-corrected bootstrap confidence intervals (CI) with 1000 draws [53]. We used Mplus (version 6.11; Muthén & Muthén, Los Angeles, CA, USA) to calculate mediation models.

We then employed correlations to examine the relationship of gender, age, and socioeconomic status to hand-washing frequency and intentions at baseline. Multivariate analyses of variance (MANOVAs) were used to examine the interaction between intervention group and moderator effects on hand-washing frequency and intentions (combined) at the 4-week follow-up. Longitudinal subgroup analyses of moderator effects could not be carried out due to the resulting small control group cell sizes (since only 1 in 6 participants were randomly assigned to the control group and to complete baseline assessments).

Since many of the variables were not normally distributed, we confirmed all analyses by equivalent nonparametric tests, which gave virtually identical results. Finally, we examined the increase in hand-washing rates and intentions in those whose level of hand-washing at baseline was less than the recommended target (ie, those scoring less than 5), as this subgroup can be considered the target population for the intervention.

## Results

### Participant Characteristics and Study Participation

**Figure 2:**
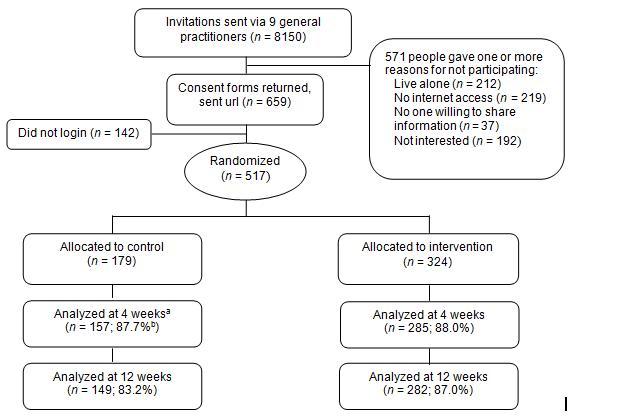
Participant flow chart for primary outcome measure (hand-washing rate).

A total of 487 people completed the primary or secondary outcome measures at either baseline or follow-up, and so were included in either the cross-sectional or longitudinal analyses. Figure 2 shows the flow of participants for the primary outcome measure. Initial uptake was low (517/8150, 6.3% of those invited underwent random allocation) and few people explained their reasons for nonparticipation. However, follow-up rates were good, with 157/179 (87.7%) control and 285/324 (88.0%) intervention group participants responding to the primary outcome measure at 4 weeks. Receipt of the intervention once allocated was also relatively good. Of the 324 of these participants who were randomly assigned to the intervention, 251 (77.5%) progressed to the second session, 219 (67.6%) completed three sessions, and 188 (58.0%) completed all four sessions.

The free hand gel was collected by 170/324 (52.5%) eligible participants. Those who collected hand gel were substantially more likely to report using hand gel at 4 weeks (*t*_272_ = 3.19, *P* = .002, *d* = 0.39), but as the mean frequency of hand gel use was only around 6 times a week this did not result in significantly higher rates of daily hand-washing (*t*_283_ = 1.36, *P* = .18, *d* = 0.16). In the intervention group, hand-washing at 4 weeks was associated with total time spent using the intervention (*r* = .23, *P* = .002) and number of sessions accessed (*r* = .21, *P* < .001).

Table 2 shows baseline characteristics in the intervention and control groups. There were no significant group differences at baseline (*P* > .10 for all comparisons). Nearly two-thirds of the sample were women, and the age range was 22 to 82 years. Among those for whom baseline hand-washing rates were assessed, 46.4% (189/407) of participants reported already achieving the recommended target of hand-washing at least 10 times a day.

Participants were excluded from these analyses only if they did not complete the primary outcome measure (hand-washing) at follow-up. Note that the sample analyzed longitudinally differs (see Table 3), as it includes those with missing data at follow-up (using imputation methods; see Statistical Analysis section) but not those allocated to the control group without baseline assessment. ^b^ Percentage of those randomly assigned to the group that were analyzed.

**Table 2:** Participant characteristics at baseline in the intervention and control groups^a^

Characteristic	Intervention (n = 336)	Control (n = 181)	Total (n = 517)
Number of women (men)	213 (123)	117 (64)	330 (187)
Age (years)	49.17 (11.02)	50.94 (12.05)	49.76 (11.40)
Socioeconomic deprivation score	9.04 (6.13)	9.39 (6.88)	9.17 (6.41)
Perceived risk	5.05 (1.62)	4.77 (1.64)	4.99 (1.63)
Hand-washing frequency	4.08 (1.05)	4.01 (1.13)	4.06 (1.07)

^a^Figures are mean (SD) except where stated.

While the range of socioeconomic status observed was quite broad (1.10 to 45.10), the sample was highly skewed toward higher status, with a median of 7.87 and an interquartile range of only 5.24–11.02. Consequently, for analyses of the effects of socioeconomic status we compared those with a score less than 12 versus those with scores ranging from 12 to 45. The median risk score in the sample was 5, and so for analyses of the effects of risk we compared those with a score of 5 or more (indicating some agreement that they were likely to catch pandemic flu) with those with scores below 5.

### Intervention Effects

As predicted, hand-washing rates were higher postintervention in the intervention than in the control group; the key comparison of hand-washing rates and intentions in the control and intervention groups was highly significant (*P* < .001) for both measures, at 4 weeks and at 12 weeks (see Table 3). Hand-washing rates were also significantly higher in the intervention group than in the control group with baseline measurement (*t*_360_ = 2.28, *P* = .02, *d* = 0.31; mean group difference = 0.30, 95% CI, 0.04–0.55), although the effect size of the intervention was somewhat greater than in those without baseline measurement (*t*_363_ = 3.41, *P* = .001, *d* = 0.45; mean group difference = 0.43, 95% CI, 0.67–0.18).

**Table 3:** Between-group comparisons of hand-washing frequency and hand-washing intentions at baseline, 4 weeks, and 12 weeks^a^

Variable	Control group		Intervention group		Difference between groups, mean (95% CI^b^)	Effect size, Cohen *d*
	n	Mean (SD)	n	Mean (SD)		
Hand-washing at baseline	91	4.01 (1.13)	316	4.08 (1.05)	0.06 (–0.20 to 0.33)	0.06
Hand-washing at 4 weeks	157	4.04 (0.86)	285	4.40 (0.86)	0.36 (0.17 to 0.55)	0.42
Hand-washing at 12 weeks	154	4.12 (1.10)	282	4.45 (0.82)	0.33 (0.13 to 0.53)	0.34
Intentions at baseline	87	4.93 (1.67)	310	5.23 (1.57)	0.30 (–0.09 to 0.70)	0.19
Intentions at 4 weeks	142	4.96 (1.71)	270	6.13 (1.18)	1.17 (0.85 to 1.48)	0.80
Intentions at 12 weeks	134	4.96 (1.68)	252	6.06 (1.21)	1.11 (0.79 to 1.43)	0.75

^a^ Hand-washing was scored from 1 (0–2 times a day) to 5 (≥10 times a day). Intentions were scored from 1 (strongly disagree) to 7 (strongly agree). Since these analyses were not baseline adjusted, sample size varied depending on response rates at follow-up. Baseline group comparisons were not significant. All group comparisons at 4 weeks and 12 weeks were significant at *P* < .001.

^b^ Confidence interval.

Longitudinal mixed-effects regression models (see Table 4) confirmed that hand-washing intentions increased from baseline to 4 weeks to a greater extent in the intervention than in the control group (time × group interaction *F*_1,375.4_ = 11.71, *P* = .001). There was also greater improvement in the theory of planned behavior cognitions in the intervention than in the control group, chiefly due to improvement in attitude in the intervention group (*F*_1,382.2_ = 14.91, *P* < .001); the effect of the intervention on subjective norm did not reach significance (*F*_1,357.9_ = 2.23, *P* = .14) and group differences in change in perceived behavioral control were negligible (*F*_1,360.8_ = 0.99, *P* = .32) (see Table 4).

**Table 4:** Change in theory of planned behavior cognitions from baseline to 4 weeks in the control and intervention groups^a^

Variable	Baseline, mean (SD)		4-week follow-up, mean (SD)	
	Control	Intervention	Control	Intervention
Intentions	4.93 (1.67)	5.23 (1.57)	5.05 (1.68)	6.00 (1.23)
Attitude	5.71 (1.28)	5.73 (1.97)	5.85 (1.11)	6.28 (0.78)
Subjective norm	4.99 (1.77)	5.15 (1.60)	5.27 (1.62)	5.66 (1.31)
Perceived behavioral control	6.11 (1.50)	6.21 (1.35)	6.47 (0.81)	6.45 (1.09)

^a^ These analyses were carried out only in those who completed measures of baseline intentions (control n = 87; intervention n = 310). All constructs were scored from 1 (strongly disagree) to 7 (strongly agree).

### Mediation of Effects on Behavior by Cognitions

As intentions and attitudes (but not subjective norms and perceived behavioral control) were changed by the intervention, we used mediation models to test whether the intervention effect might be mediated by changes in intentions or attitudes. Results showed significant positive indirect effects of the intervention on change in hand-washing via intentions (coefficient = .15, 95% CI, .08–.26) as well as attitudes (coefficient = .16, 95% CI, .09–.26). The direct effect of the intervention on change in hand-washing dropped to nonsignificance when cognitions were included in the models, in both cases.

### Effects of Moderator Variables

At baseline, female gender was associated with higher levels of hand-washing (*r* = .34, *P* < .001) and intentions (*r* = .36, *P* < .001). There were no associations between age and hand-washing frequency (*r* = .02, *P* = .69) or intentions (*r* = –.01, *P* = .82). Greater socioeconomic deprivation was associated with slightly higher levels of hand-washing frequency (*r* = .12, *P* = .02) and intentions (*r* = .12, *P* = .01). Greater perceived risk was also associated with higher levels of hand-washing frequency (*r* = .25, *P* < .001) and intentions (*r* = .37, *P* < .001).

We then examined whether significant baseline predictors of hand-washing frequency and intentions moderated the effectiveness of the intervention. MANOVA revealed a main effect of gender on hand-washing frequency and intentions (*F*_2,407_ = 12.61, *P* < .001; partial *η* *2* = .058) but no interaction with intervention group (*F*_2,407_ = 0.30, *P* = .74; partial *η* *2* = .001). There was also a main effect of perceived risk on hand-washing frequency and intentions (*F*_2,331_ = 14.31, *P* < .001; partial *η* *2* = .080) but no interaction with intervention group (*F*_2,331_ = 0.69, *P* = .502; partial *η* *2* = .004). There was no effect of socioeconomic status on hand-washing frequency and intentions (*F*_2,407_ = 0.67, *P* = .51; partial *η* *2* = .003) and no interaction with intervention group (*F*_2,407_ = 0.35, *P* = .70; partial *η* *2* = .002).

Although the study was not powered to test for differences between subgroups, inspection of Table 5 and Table 6 shows a trend toward higher hand-washing rates and intentions in the intervention group in both men and women, those of higher and lower socioeconomic status, those with higher and lower levels of perceived risk, and those whose level of hand-washing at baseline was less than that recommended (see Table 5 and Table 6). There was an interaction between intervention group and baseline hand-washing rates for both hand-washing frequency (*F*_1,358_ = 11.95, *P* = .001, partial *η* *2* = .032) and intentions (*F*_1,358_ = 11.95, *P* = .001, partial *η* *2* = .032), confirming that improvement as a result of the intervention was greater in those with lower hand-washing levels. This was due partly to ceiling effects, since none of those already reporting hand-washing at the recommended rate at baseline could improve on that measure (although some could on the hand-washing intentions measure).

**Table 5:** Moderator effects on hand-washing frequency in the intervention and control groups at 4-week follow-up

Variable		Control group		Intervention group	
		n	Mean (SD)	n	Mean (SD)
**Gender**					
	Male	53	3.77 (1.03)	101	4.10 (0.10)
	Female	104	4.17 (1.01)	184	4.57 (0.73)
**Socioeconomic status**					
	Lower deprivation	111	3.99 (1.07)	215	4.39(0.86)
	Higher deprivation	46	4.15 (0.92)	70	4.43 (0.86)
**Perceived risk**					
	Lower risk	35	3.77 (1.14)	93	4.10 (1.02)
	Higher risk	44	4.32 (0.91)	185	4.58 (0.69)
**Baseline hand-washing**					
	Lower rate	42	3.40 (0.96)	146	4.08 (0.95)
	Higher rate	39	4.79 (0.52)	135	4.79 (0.51)

**Table 6:** Moderator effects on hand-washing intentions in the intervention and control groups at 4-week follow-up

Variable		Control group		Intervention group	
		n	Mean (SD)	n	Mean (SD)
**Gender**					
	Male	50	3.77 (1.03)	92	5.01 (1.41)
	Female	92	4.17 (1.01)	178	4.57 (0.73)
**Socioeconomic status**					
	Lower deprivation	101	4.94 (1.69)	204	6.06 (1.19)
	Higher deprivation	41	5.02 (1.78)	66	6.34 (1.12)
**Perceived risk**					
	Lower risk	32	4.67 (1.71)	88	5.72 (1.43)
	Higher risk	41	5.63 (1.25)	175	6.34 (0.92)
**Baseline hand-washing**					
	Lower rate	38	4.53 (1.66)	136	5.81 (1.40)
	Higher rate	37	5.89 (1.06)	130	6.47 (0.79)

## Discussion

Participants given access to the Web-based intervention had higher levels of reported hand-washing frequency and intentions for frequent hand-washing in the future than those in the control group (with or without baseline measurement). This higher level of hand-washing was maintained at 12 weeks, as predicted by our primary hypotheses. These findings provide encouraging evidence that hygienic behavior may be effectively promoted by a theory-based online intervention. The medium effect sizes for reported behavior that we observed were larger than the average for Web-based interventions [54] and similar to other online interventions based on the theory of planned behavior [55]. We predicted and observed relatively modest changes in hand-washing, which is a largely habitual behavior, but these changes would nevertheless be sufficient to be valuable if replicated across much of the population. At the time of this study, participants in both groups had been exposed to considerable media and government coverage of the need for hand hygiene during the pandemic, suggesting that our intervention could usefully add to existing public health campaigns.

Moderator analyses did not reveal any significant differences in the effectiveness of the intervention for those of high and low socioeconomic status, men and women, and those with higher and lower levels of perceived risk of infection. These analyses are important in terms of establishing the suitability of the intervention for rolling out to the general population [19], and although the study was not powered to detect subgroup differences, it is reassuring that we observed a trend toward higher hand-washing rates in all the intervention subgroups. There was no evidence that socioeconomic status had a negative impact on hand-washing, but our intervention was unable to eliminate differences in hand-washing rates associated with gender and perceived risk of infection; additional efforts may be needed to elevate hand-washing rates among men. In the event of a serious pandemic it is likely that both perceived risk and motivation to wash their hands will increase throughout the population [12,14,25].

Our planned examination of whether the intervention changed theory of planned behavior cognitions revealed substantial effects on intentions and attitudes. Although mediation model tests cannot prove causation, the findings of the mediation analyses indicated that the data were consistent with a mediation model where attitudes and intentions mediated the effects of the intervention on behavior. However, we observed no change in subjective norms or perceived behavioral control. Perceived behavioral control was already high at baseline and so a ceiling effect likely limited the potential for the intervention to increase it further. However, there was scope for improvement in subjective norms, and since social norms are an important influence on hand-washing [56], these findings suggest it might be advisable to supplement our intervention with more effective methods of changing the perceived social desirability of hand-washing.

A major limitation of our study is that only self-reported hand-washing could be assessed, which is likely to overestimate actual levels of hand-washing [15]. There were some indications that self-reports did not simply reflect socially desirable responding: higher rates of hand-washing were associated with objective measures of intervention use, and hand-washing with gel was related to objective measures of collecting hand gel. Moreover, although it seemed likely that our self-selected sample would have had above-average levels of motivation to wash their hands, reported hand-washing rates at baseline were actually slightly below UK rates reported during the pandemic [14]. The problem remains that self-report cannot be considered to provide a definitive test of whether behavior actually changed, but observation of hand-washing within the home in large samples is intrusive and impractical. However, the aim of this study was to estimate the behavioral effects of the intervention in preparation for a large trial of intervention effects on actual infection rates. For this purpose, it was essential to show that the intervention could at least influence self-reported intentions and behavior, as these can be considered a necessary (though not sufficient) precursor of reductions in infection transmission. The large study of infection rates will then allow us to perform a more definitive test of whether any reduction in infection rates achieved by this intervention is mediated by self-reported hand-washing.

A second major limitation was that our uptake rate was less than 1 in 10, and our sample overrepresented affluent, middle-aged women. This profile is typical of those who engage with Web-based health promotion [57] and suggests that it may be necessary to supplement Web-based interventions in order to reach all segments of the population; in particular, future research should establish the most effective interventions for reaching older people and ethnic minority groups, who are typically the worst affected in pandemics. Nonetheless, the moderator analyses provided some reassurance that the intervention should prove suitable for those socially deprived people who do access Web-based health interventions, and could provide a cost-effective means of reaching much of the population quickly in a pandemic.

A strength of this study is that it pragmatically assessed the effectiveness of the intervention, by calculating the effect size when compared with a control group without baseline assessment, but it also examined the efficacy of the intervention, by calculating the effect size when compared with a control group with baseline assessment. It was valuable to demonstrate that the intervention was successful when evaluated in both these ways, but this exploratory trial was not powered to make subgroup comparisons; it would be useful in future research to specifically test whether the somewhat lower effect size observed when the control group had received baseline assessment was indeed due to the effects of completing the baseline assessments.

In conclusion, this study provided the first demonstration of the potential value of a theory-based online intervention to promote behavior intended to reduce or slow the transmission of respiratory infection. An advantage for the pandemic context is that it is feasible and inexpensive to rapidly make available an intervention of this kind to a wide population, thus preserving resources for targeting groups that may require different types of intervention. For example, since this intervention was fully automated, it could be easily disseminated by links to frequently accessed health care websites and by advertising the website in government media campaigns providing information about coping with seasonal or pandemic influenza. However, further research is first required to determine the effects of the intervention on actual infection rates.

## Multimedia Appendix 1

Sample screen shots from PRIMIT intervention.

## Multimedia Appendix 2

Further details of intervention development and content.

## Multimedia Appendix 3

Patient information leaflet.

## Multimedia Appendix 4

PRIMIT protocol.

## Abbreviations

CI: confidence interval
MANOVA: multivariate analysis of variance
PRIMIT: PRimary care trial of a website based Infection control intervention to Modify Influenza-like illness and respiratory infection Transmission
SARS: severe acute respiratory syndrome
