# Supplementary figures and images for: Evaluation of a Web-Based Intervention to Promote Hand Hygiene: Exploratory Randomized Controlled Trial
Source: J Med Internet Res. 2011 Dec 9;13(4):e107. doi: 10.2196/jmir.1963 (PMC3278093; doi:10.2196/jmir.1963)

## Slide 1
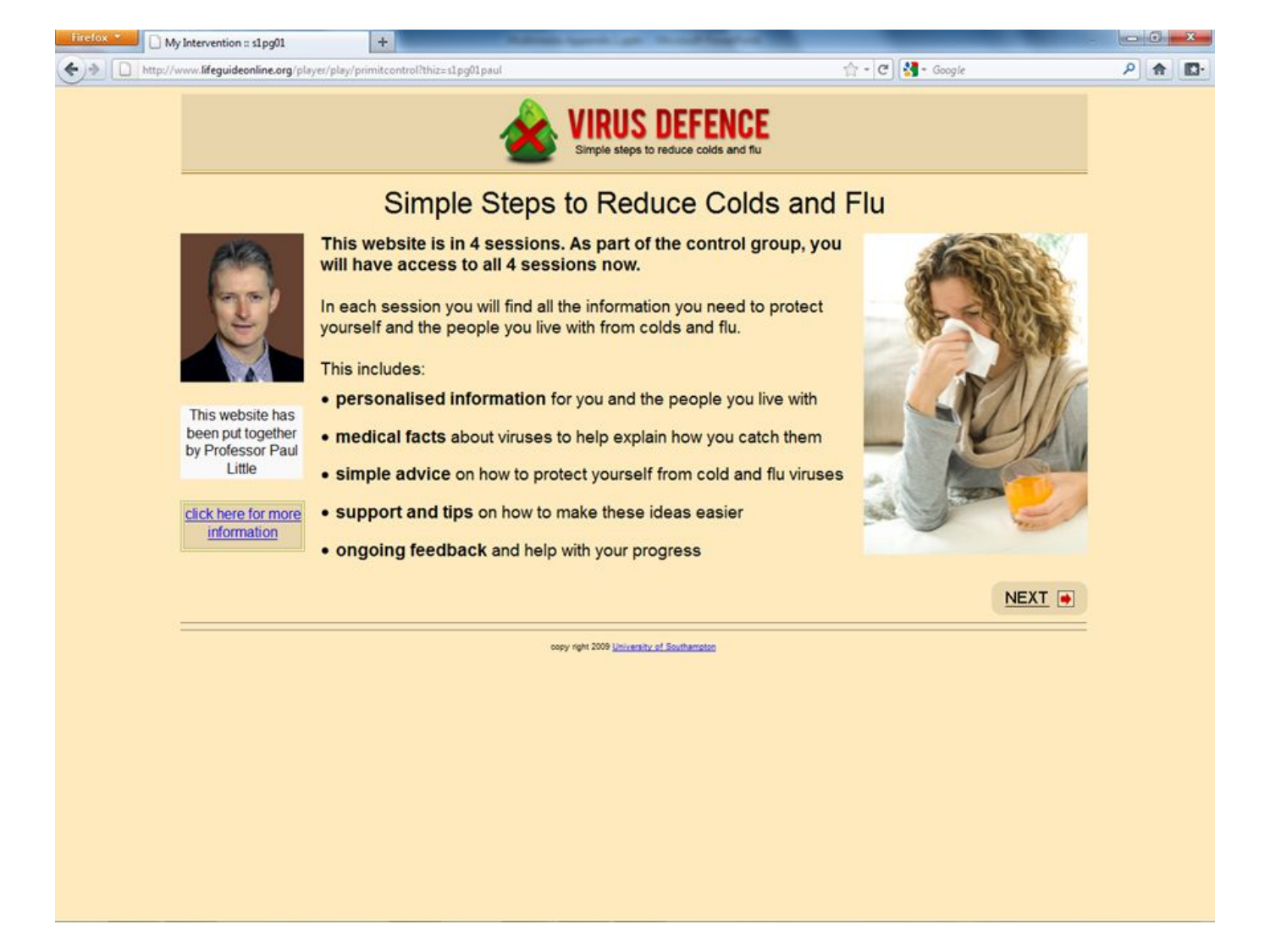

## Slide 2
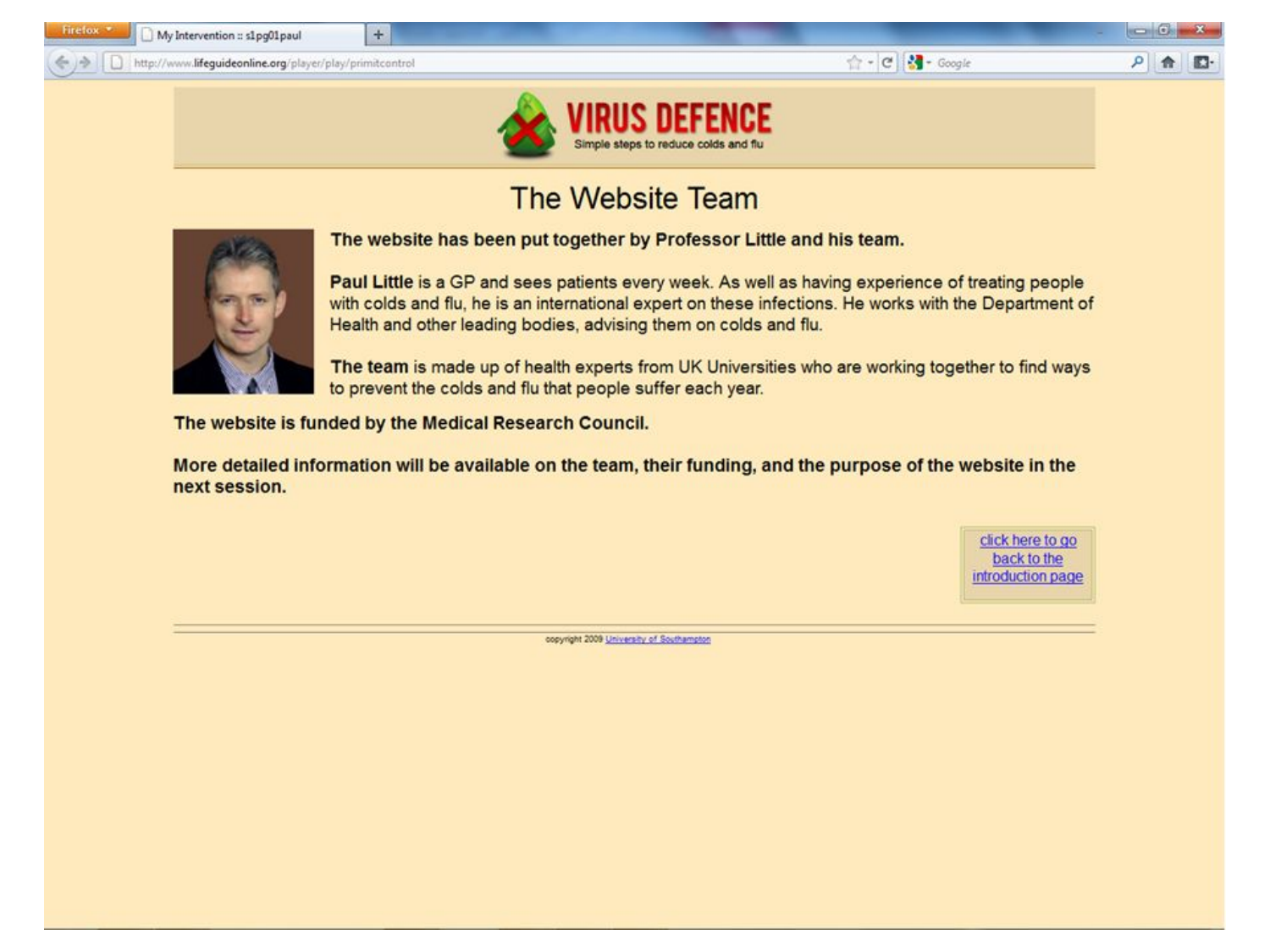

## Slide 3
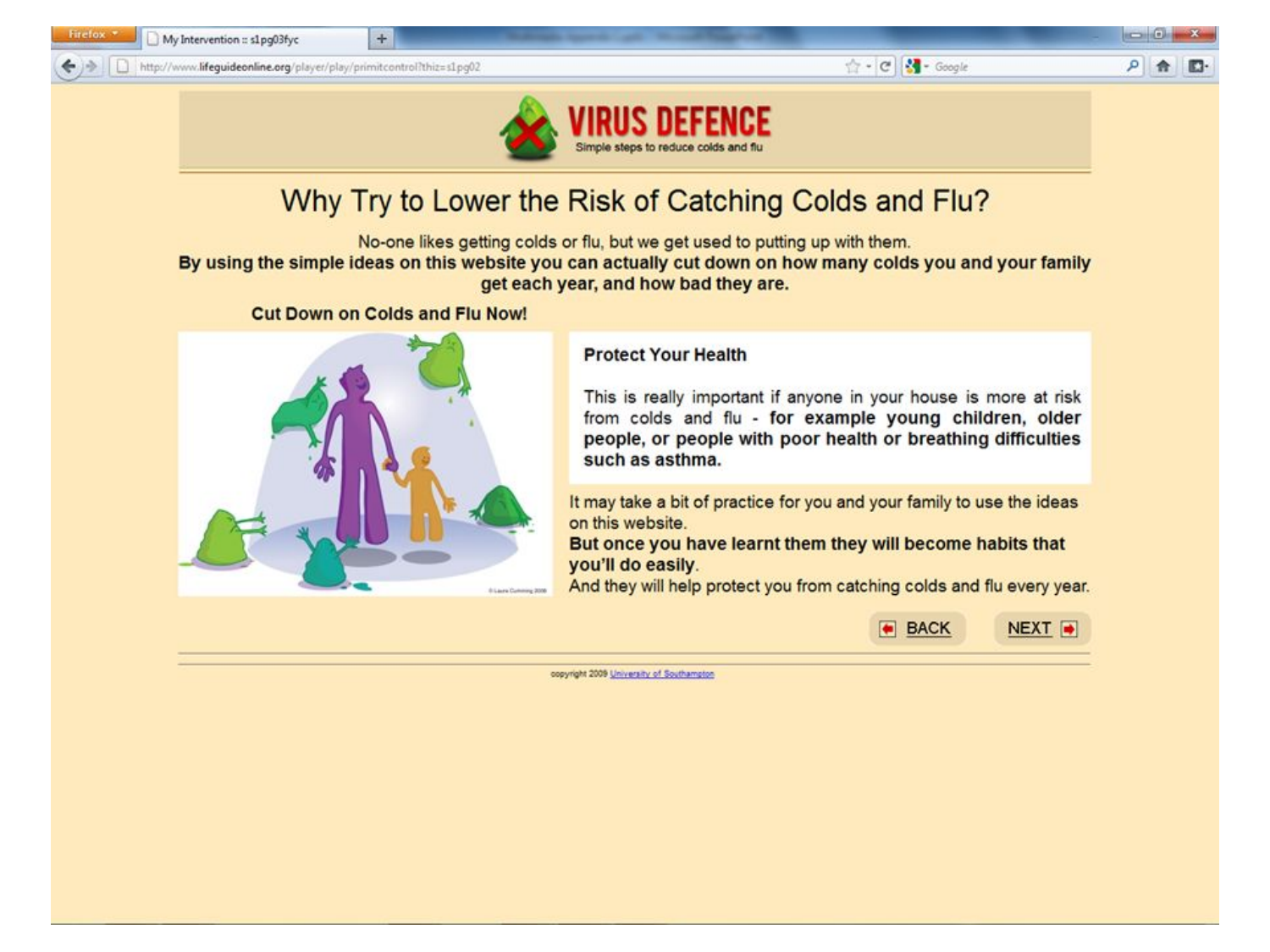

## Slide 4
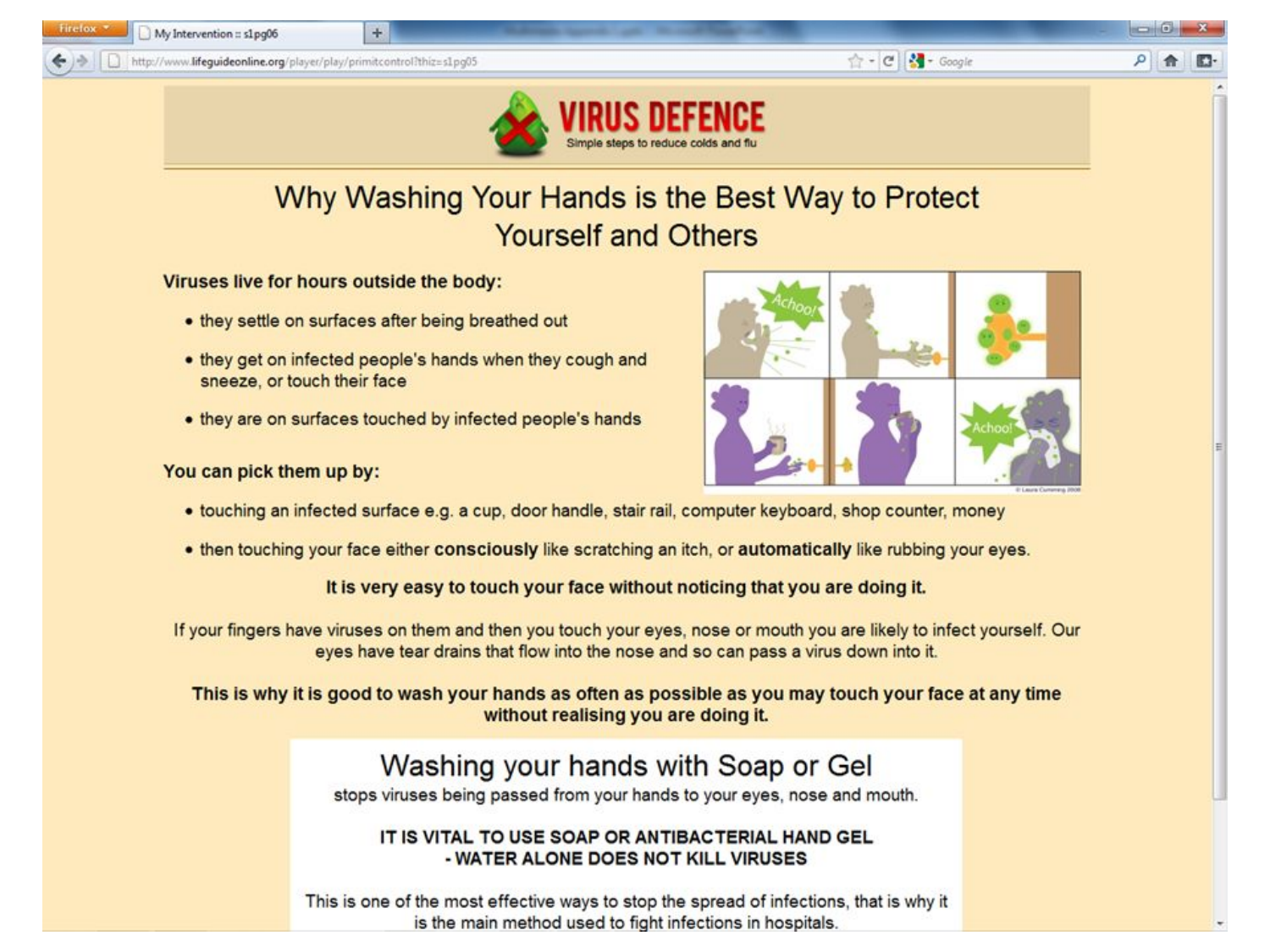

## Slide 5
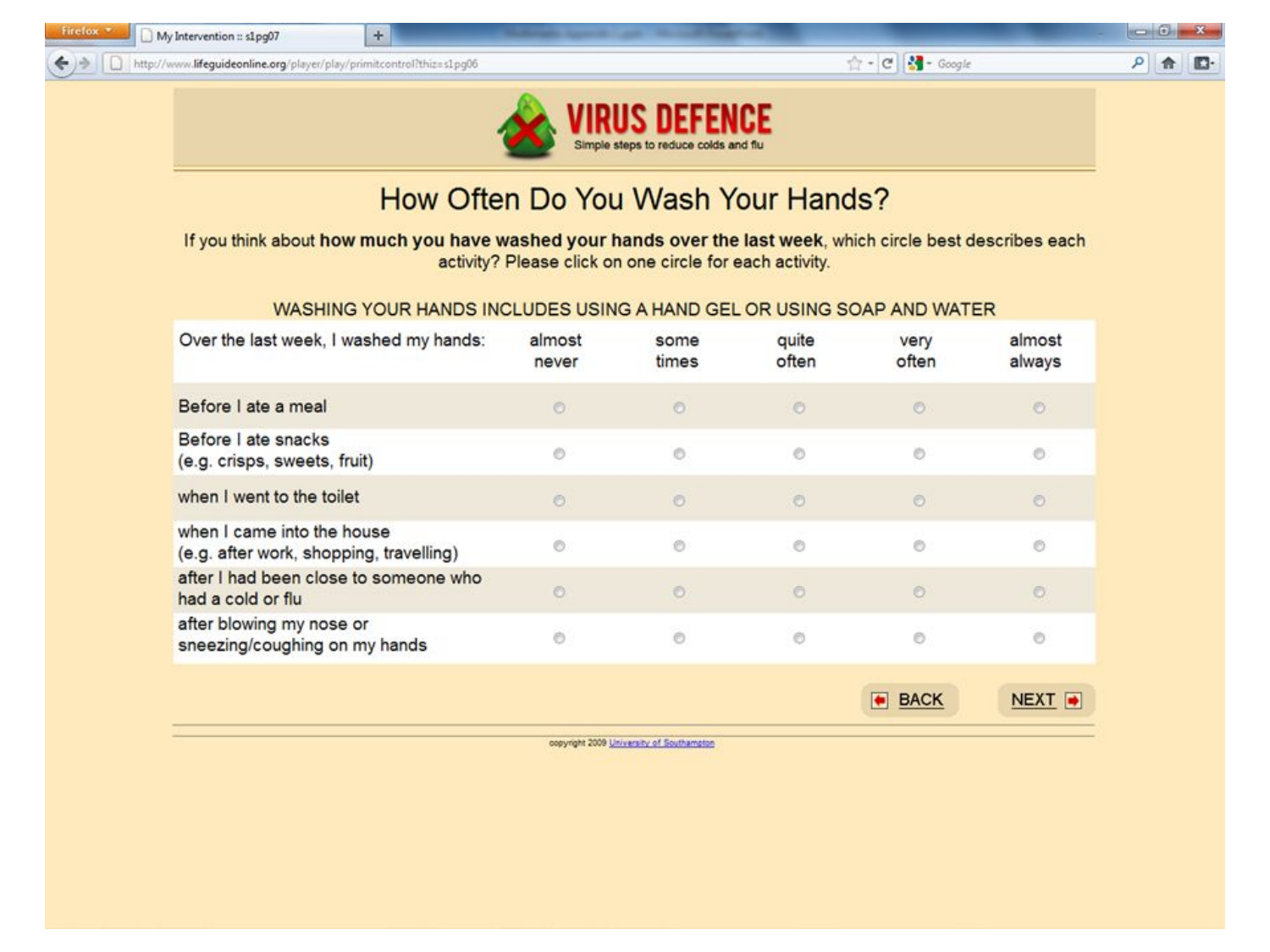

## Slide 6
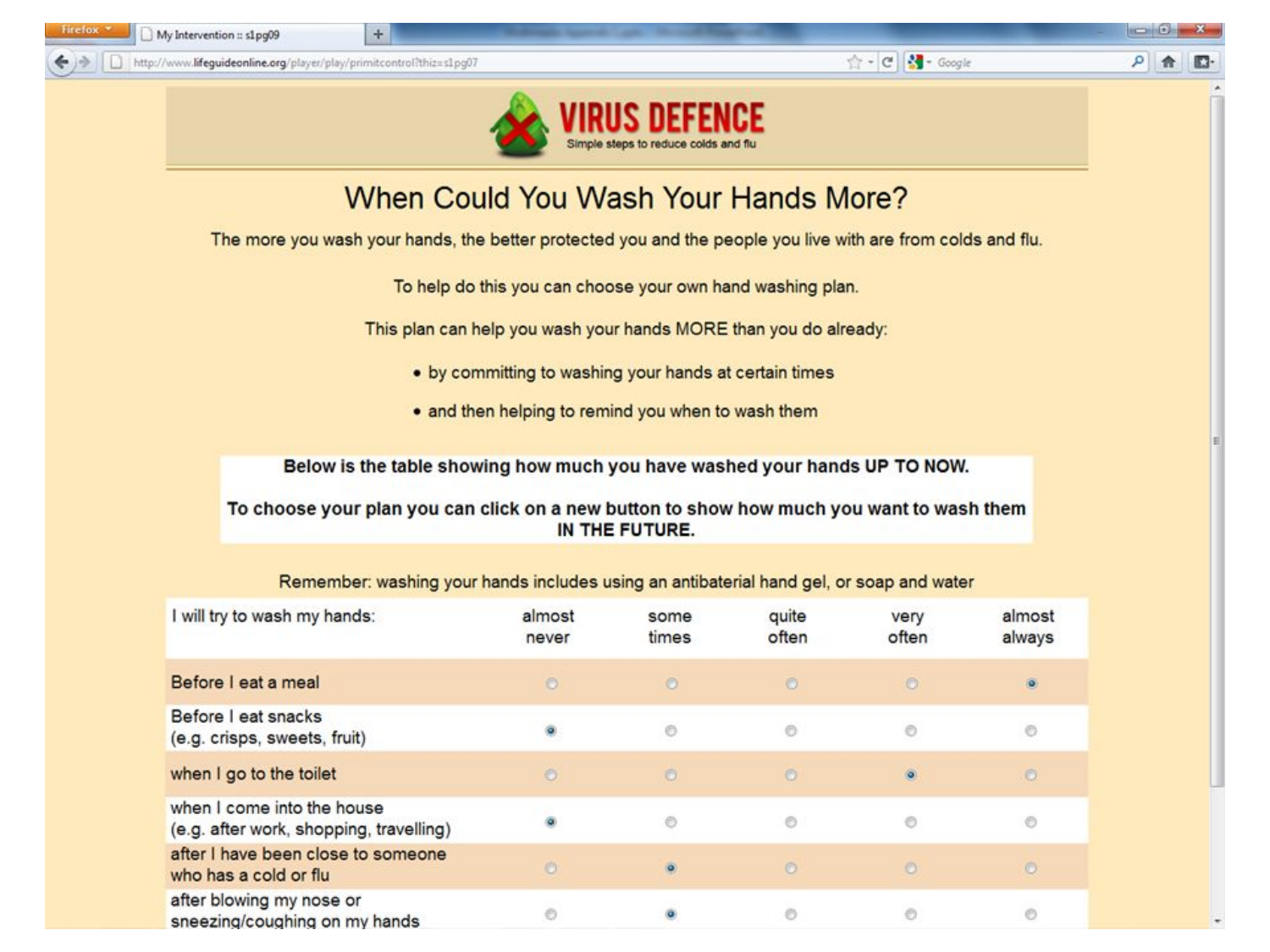

## Slide 7
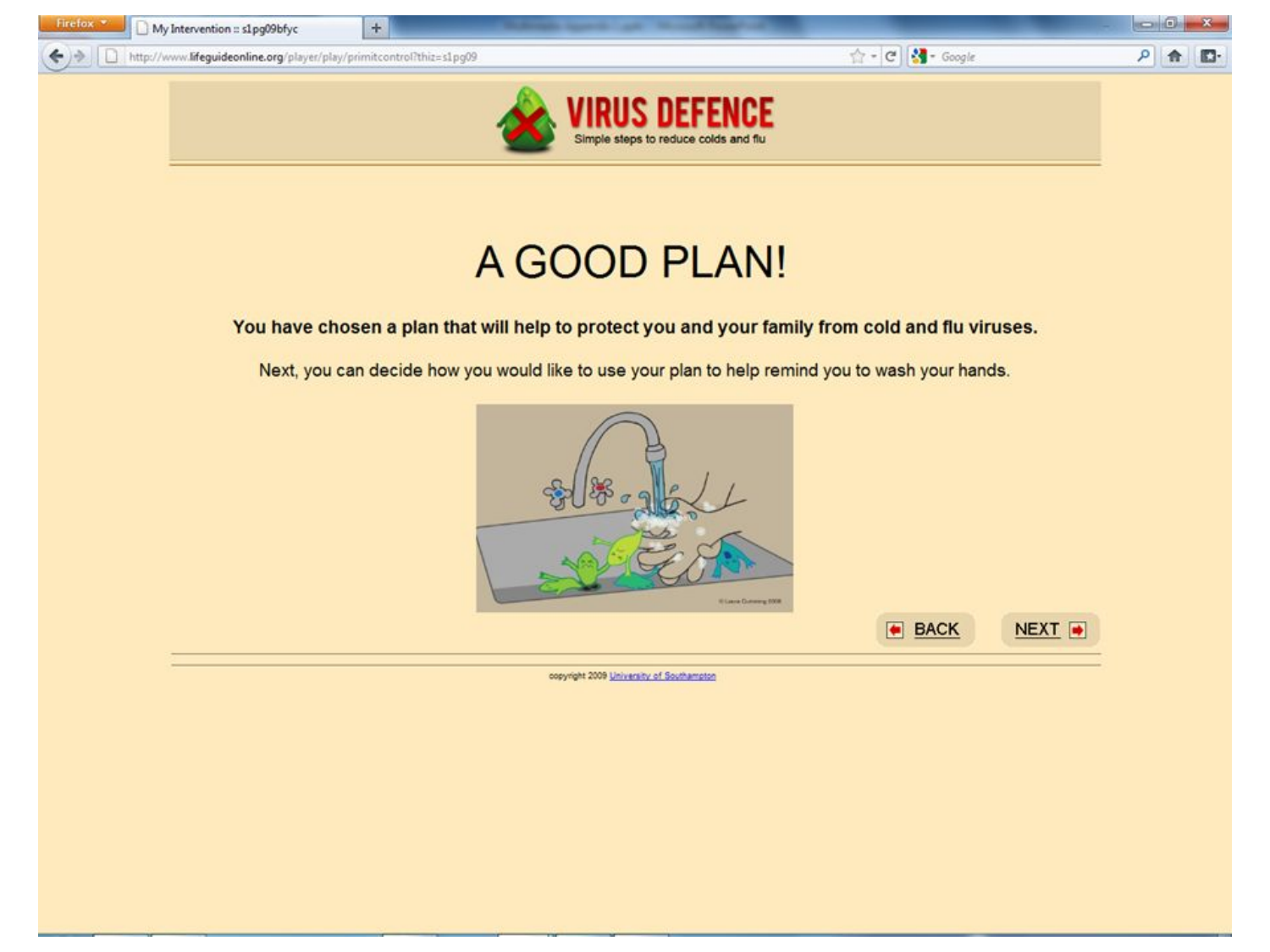

## Slide 8
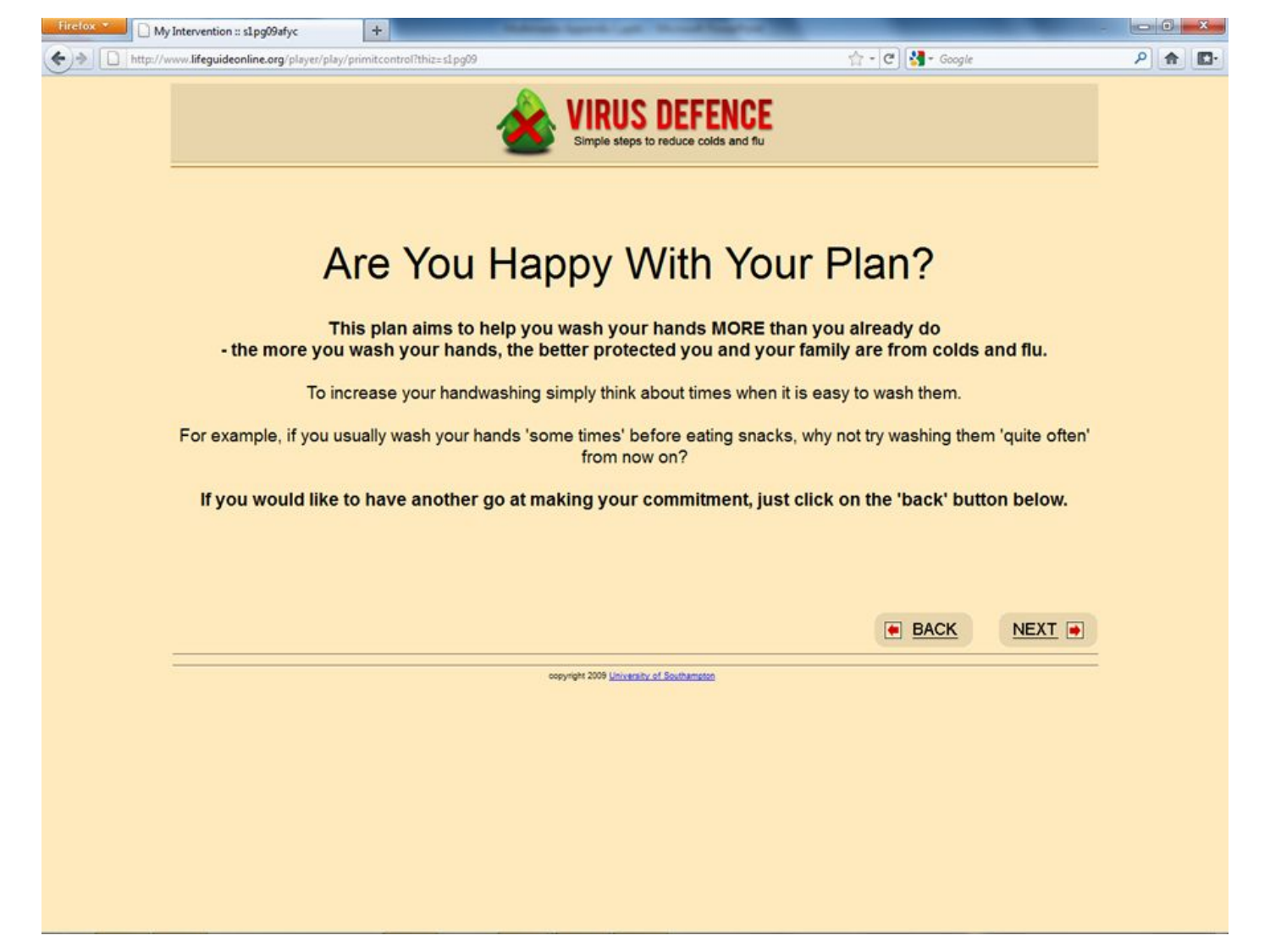

## Slide 9
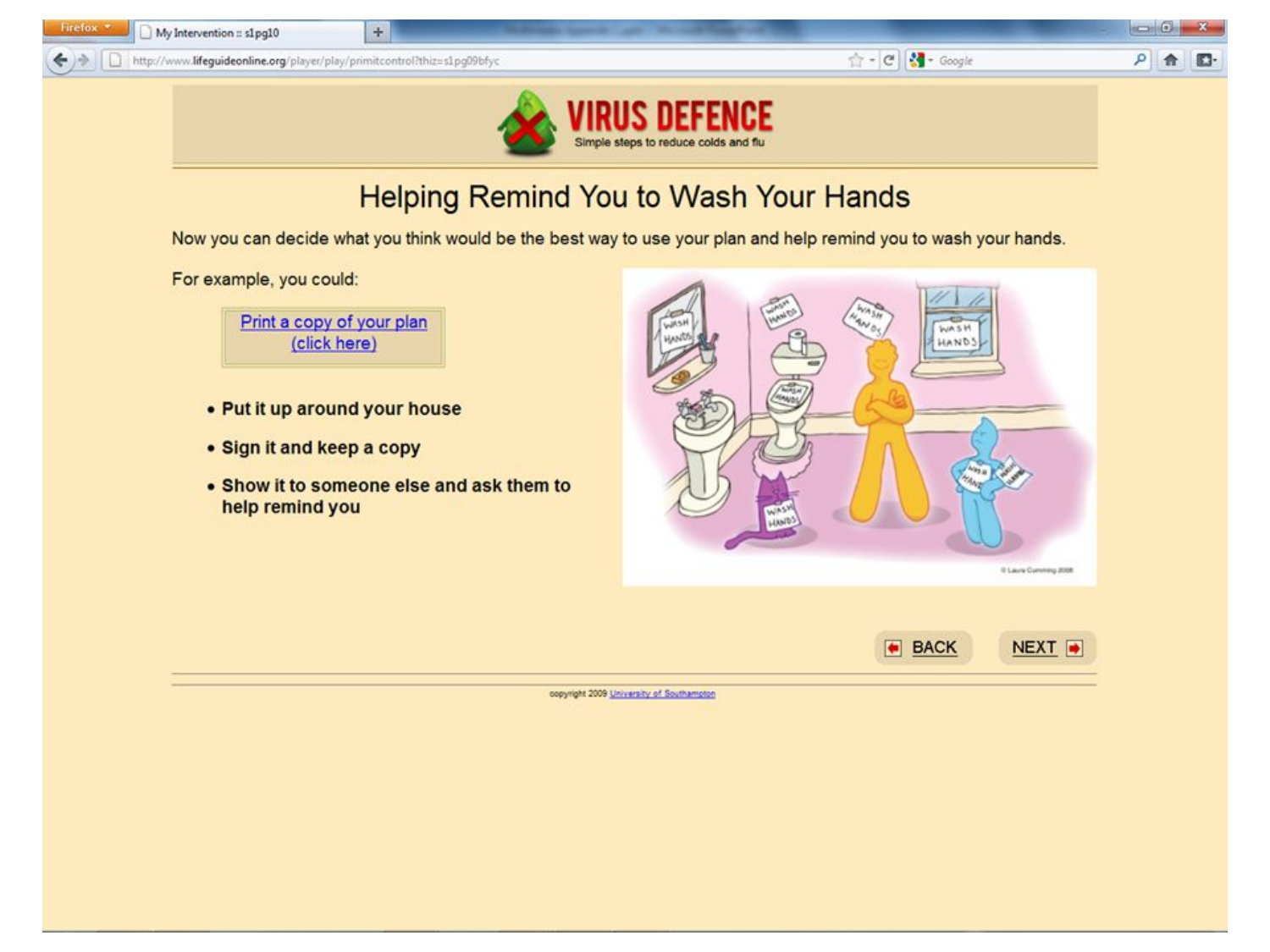

## Slide 10
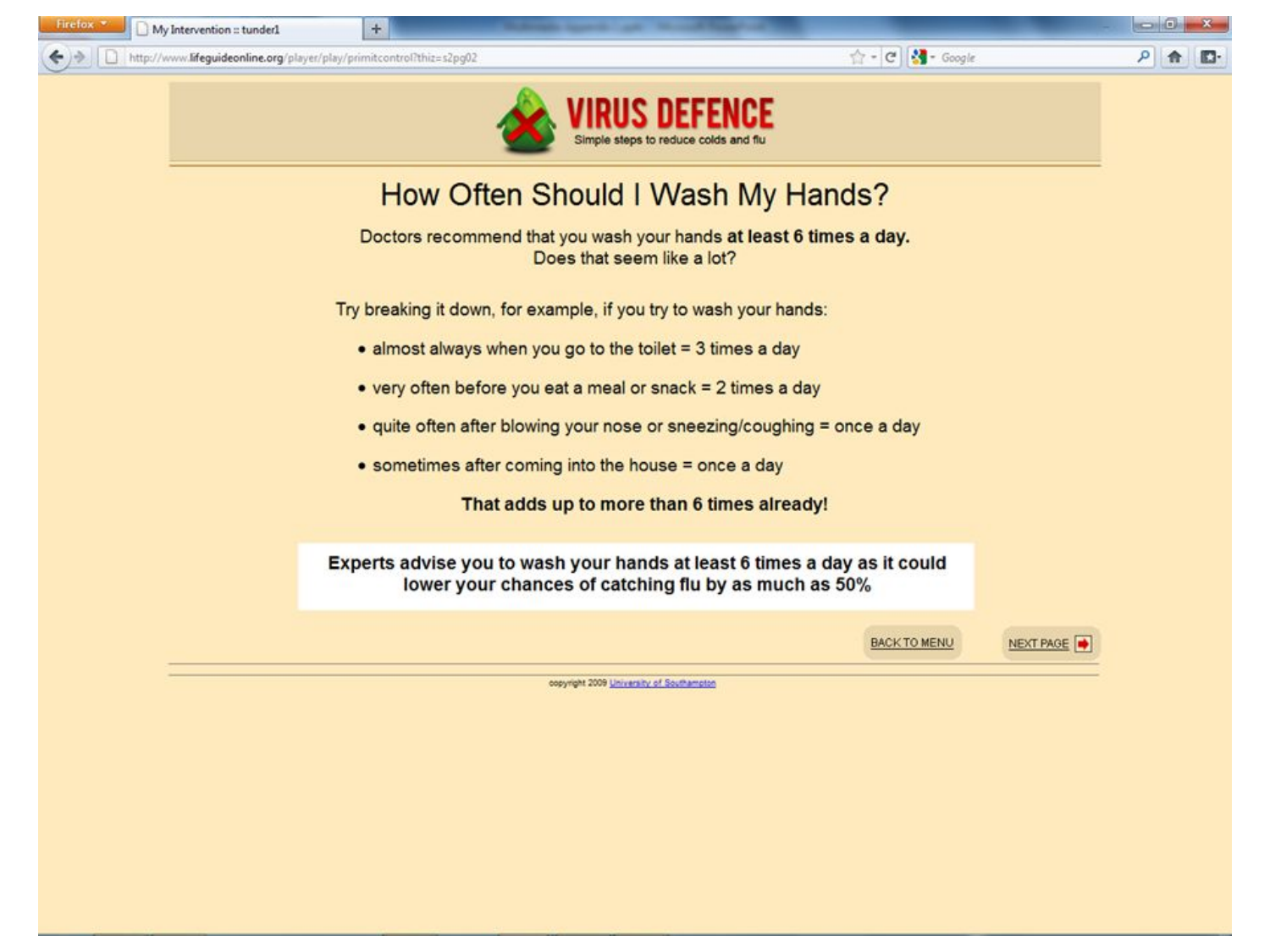

## Slide 11
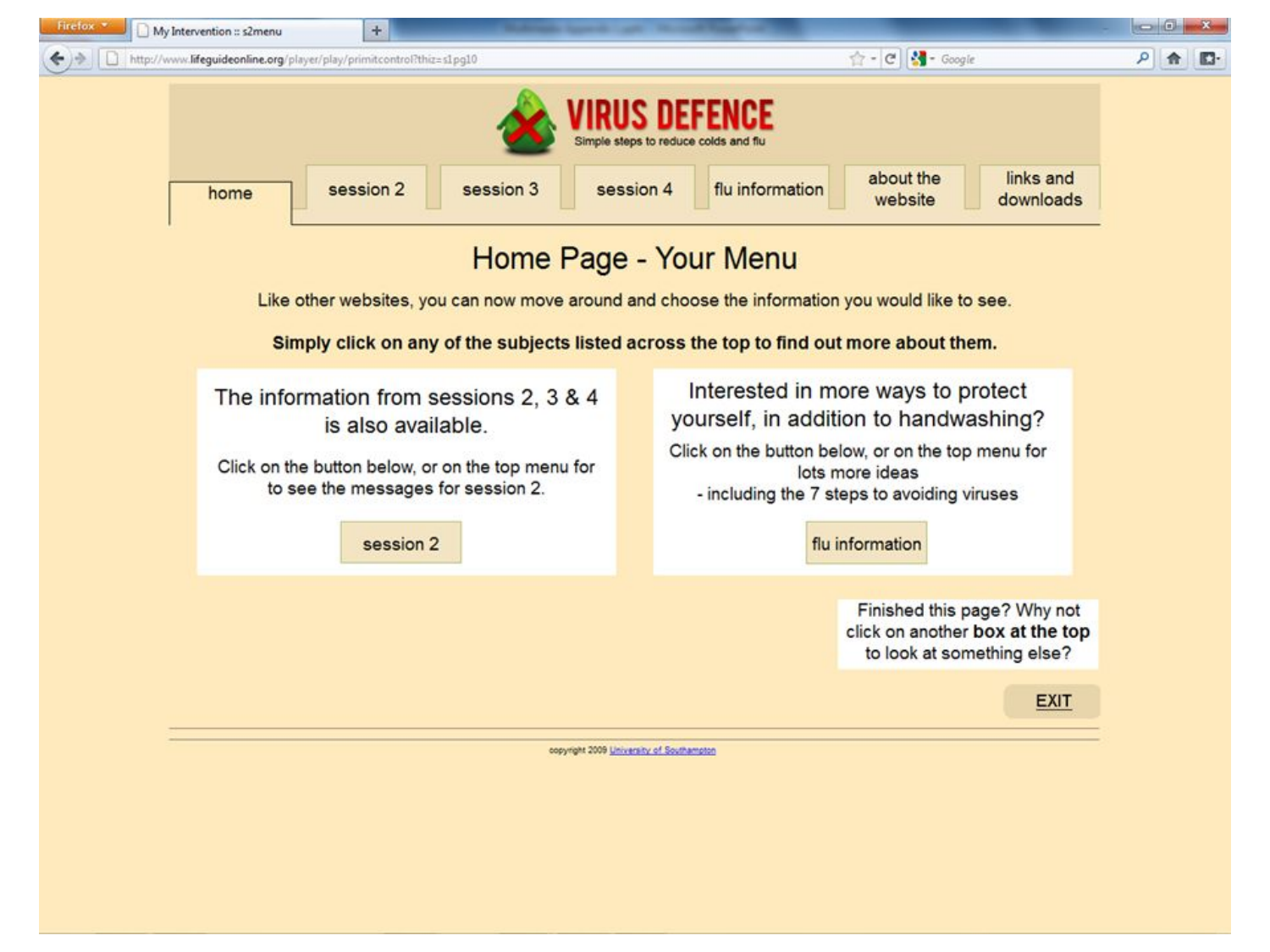

## Slide 12
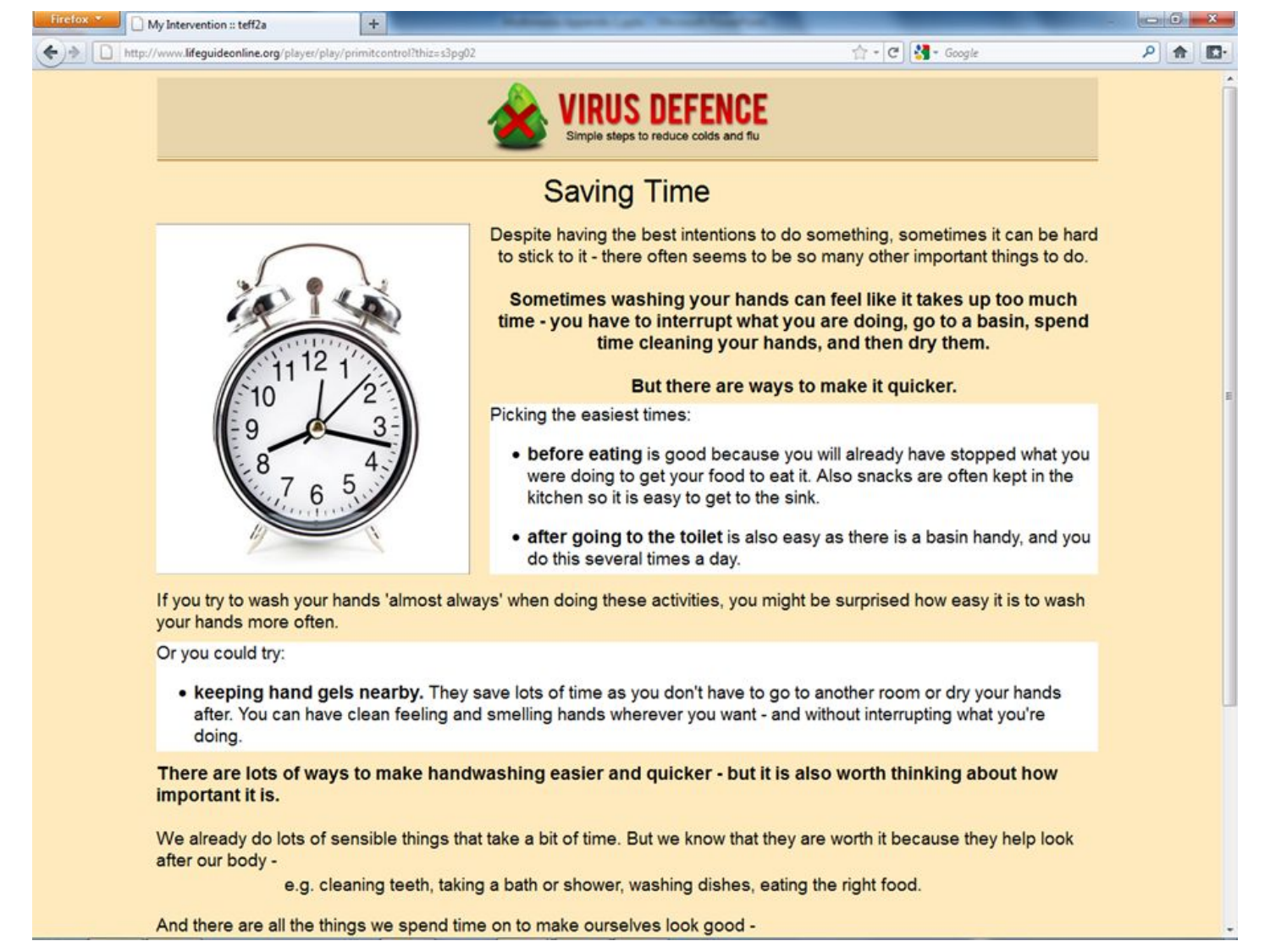

## Slide 13
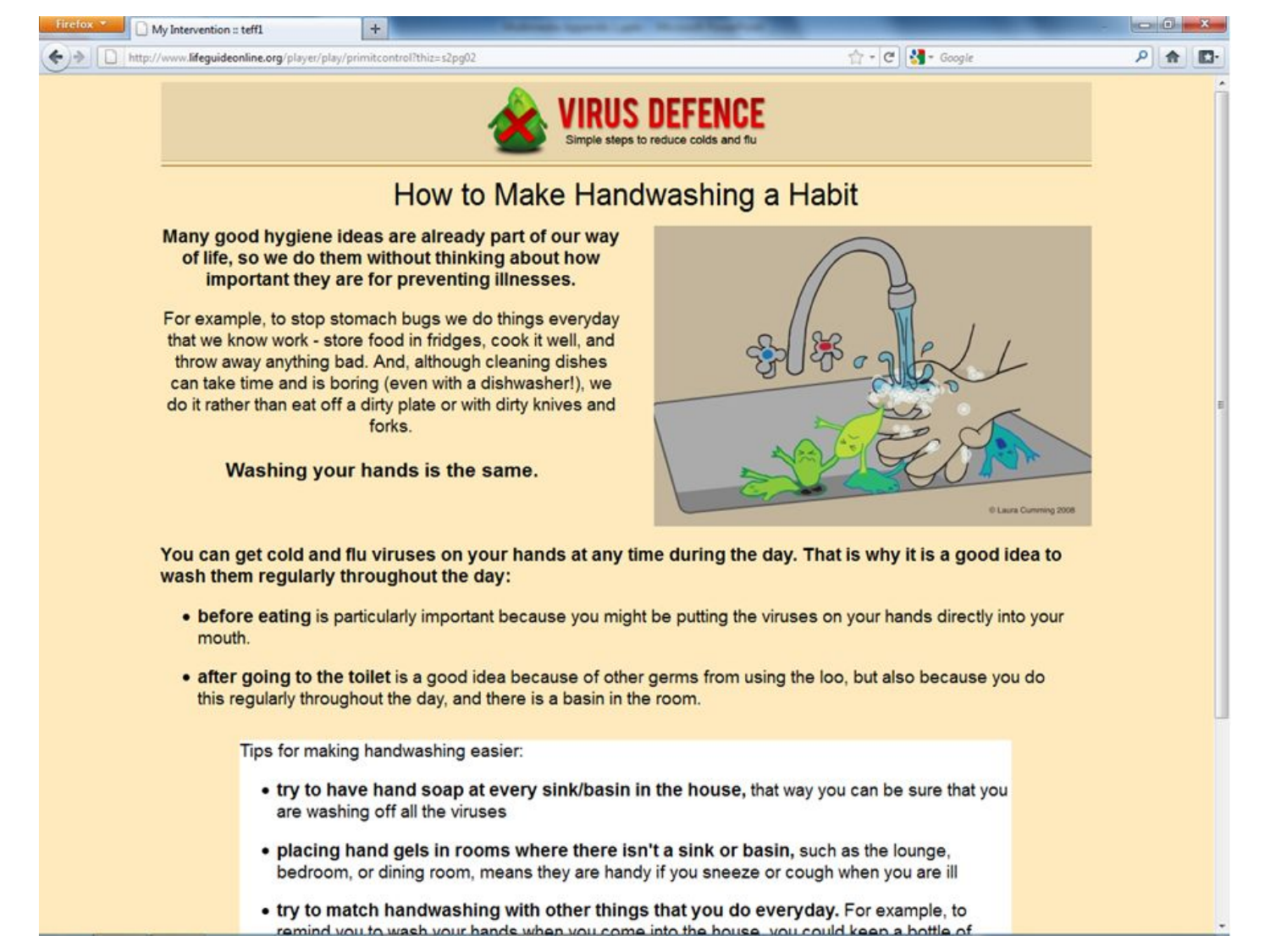

## Slide 14
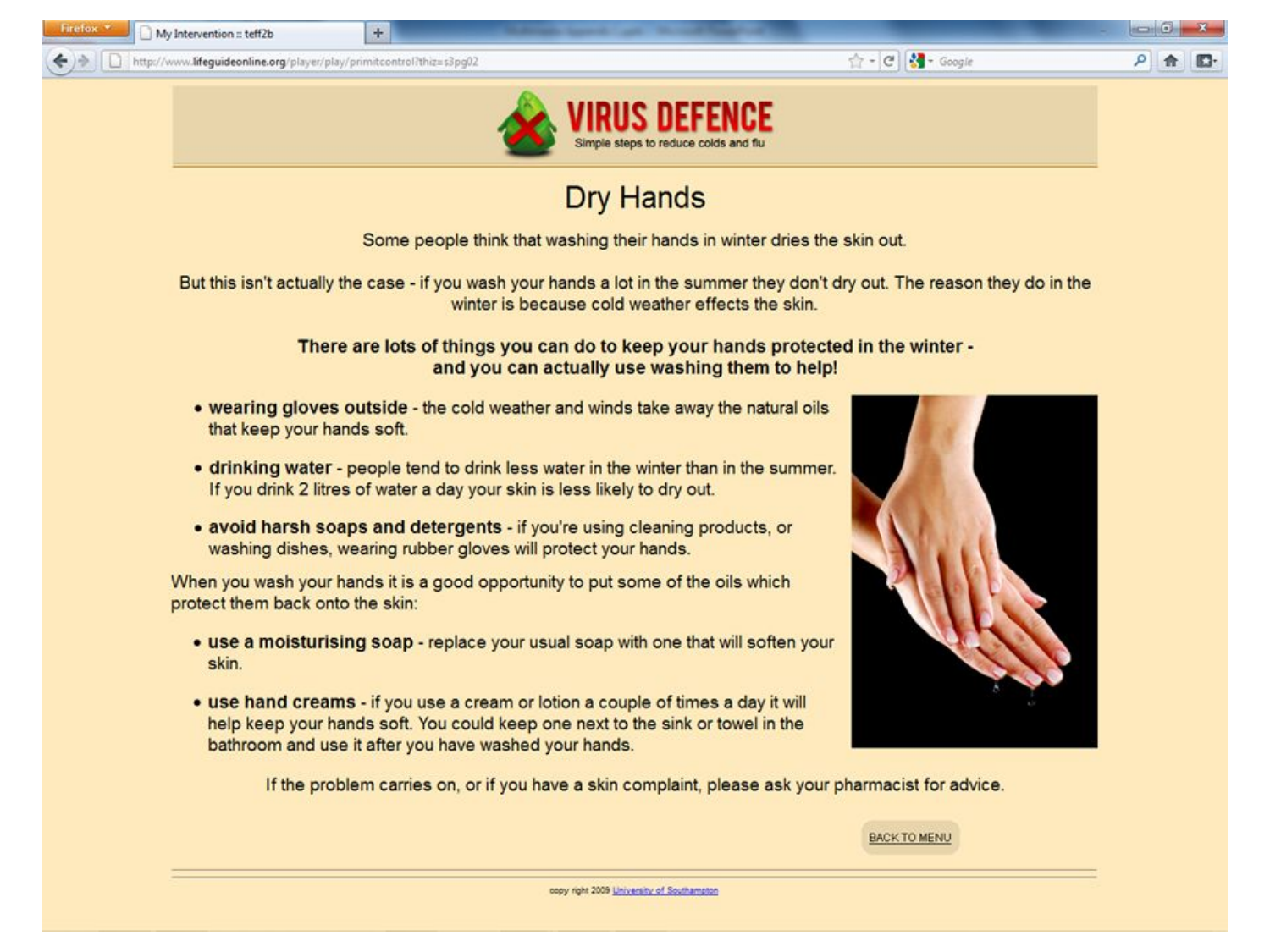

## Slide 15
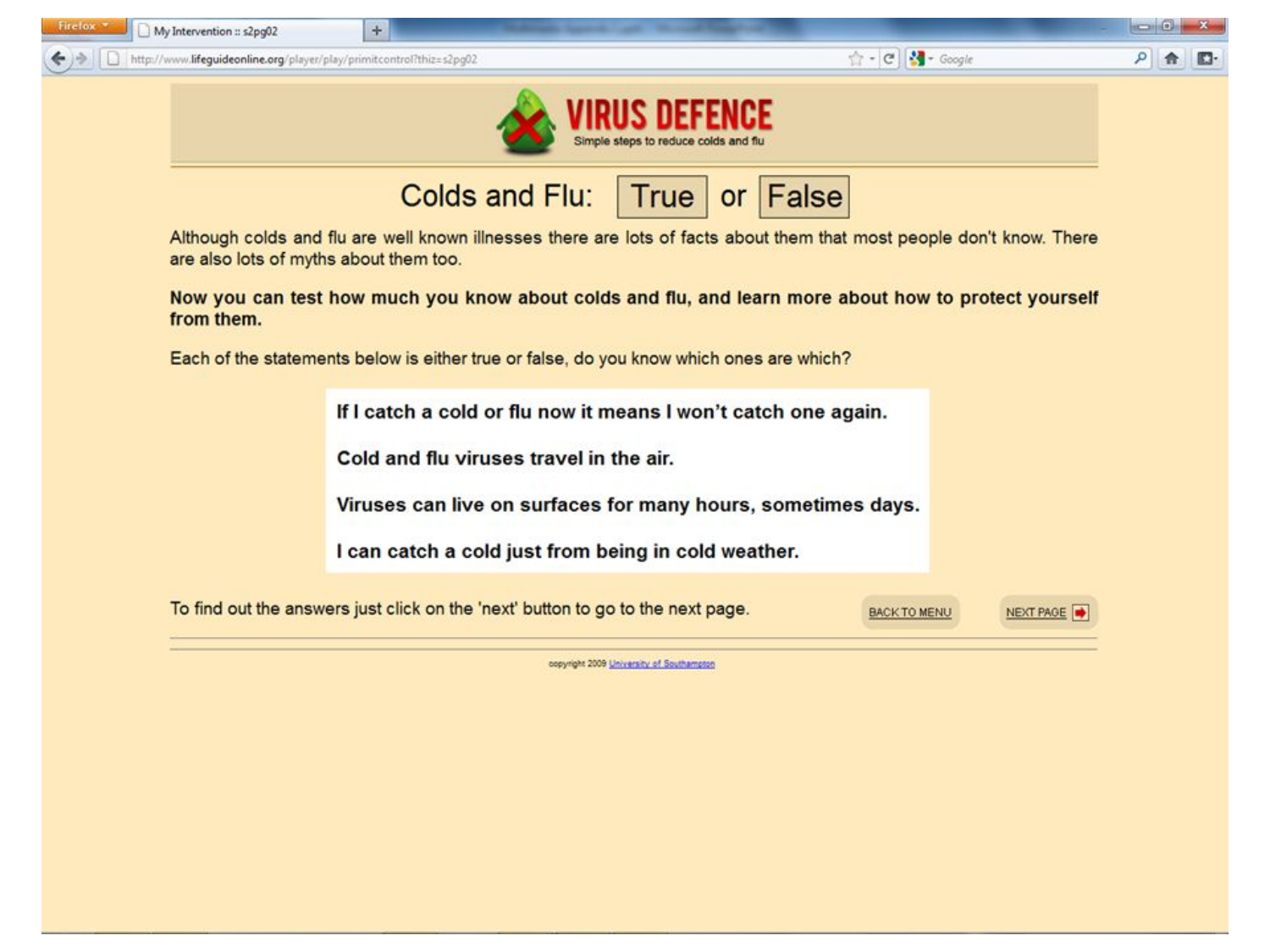

Supplement: Supplementary file 1 [file jmir_v13i4e107_app1.ppt]
