# Supplementary material for: Evaluation of a Web-Based Intervention to Promote Hand Hygiene: Exploratory Randomized Controlled Trial
Source: J Med Internet Res. 2011 Dec 9;13(4):e107. doi: 10.2196/jmir.1963 (PMC3278093; doi:10.2196/jmir.1963)
Supplement: Supplementary file 2 [file jmir_v13i4e107_app2.pdf]

Multimedia Appendix 2: Further details of intervention development and content

Intervention development

Development of intervention content was guided by the theoretical frameworks referenced in the paper, and review of existing relevant empirical literature on hand-washing in the context of seasonal and pandemic infection. From these sources, and previous experience of web-based intervention development, the first author identified likely key determinants of behavior and effective web-based methods of addressing these. The first and second author then drafted the webpages. An informal mapping process was employed to check that each of the relevant factors identified from theory and literature had been addressed (there was insufficient time to carry out an exhaustive, systematic reviewing and intervention mapping process). To check that the intervention was effectively addressing the right behaviors and determinants we also carried out four empirical studies, as follows:

1. Ascertaining the feasibility of target behaviors and influences on adherence to them [1].

Following Green and Kreuter's 'PRECEDE-PROCEED' model [2], we asked infection experts on our team to identify the behaviors most likely to be efficacious. We then carried out a qualitative study (N = 31) to determine which of these were also viewed as feasible and acceptable by our target population, and what factors would influence their adherence to the behaviors. Participants were invited to discuss their perceptions of infection transmission and likely adherence to infection control measures in both non-pandemic and pandemic contexts. Infection control measures discussed included handwashing, social distancing and cough hygiene (e.g. covering mouth, disposing of tissues immediately etc.). Thematic analysis revealed that handwashing was regarded by our participants as more feasible than cough and sneeze hygiene and more acceptable than social distancing, and identified a number of factors that were potential barriers to carrying out infection control behaviors.

2. Confirming the feasibility of target behaviors and influences on adherence to them [3].

We then employed a questionnaire study (*N* = 129) to test the key assumptions of our intervention: a) that hand-washing would be viewed as the most feasible preventive behavior; b) that the behavioral determinants we had identified were related to behavioral intentions; c) that the beliefs addressed by our intervention were related to hand-washing intentions and behavior. The questionnaire results confirmed our assumptions, including our selection of hand-washing as the most feasible target behavior, since few people felt able to successfully practice cough-sneeze hygiene while mask-wearing and social distancing were viewed as unattractive and impractical.

3. Checking whether our draft intervention materials effectively addressed the relevant beliefs of our target population [3].

Two qualitative studies comprising'think aloud' sessions and interviews were carried out to examine how 28 potential users responded to draft website materials and the prototype website, and to elicit any additional beliefs which our intervention might need to address. Thematic analysis of the qualitative data revealed important additional issues to address, such as the belief that catching minor infections is beneficial to the immune system and that conscientious hand-washing may seem impolite or obsessive. The web-pages were modified to address all feedback from these interviews, including improving the attractiveness, clarity and ease of use of the website, including more engaging formats (e.g. quizzes), as well as adding new content to address the additional issues identified.

4. Confirming the relevance of the behavioral determinants we addressed and the validity of our measures [4].

The main purpose of this study was to confirm that the behavioral determinants we had identified were related to intentions and establish the validity of our measures of behavioral intentions. Participants (N = 84) completed a self-report web-delivered questionnaire measuring intentions to engage in hand-washing and hypothesized behavioral determinants of intentions, based on the Theory of Planned Behavior and Protection Motivation Theory. In a factorial 2X2 design half the participants were first randomized to receive messages about potential negative consequences of pandemic flu (the 'high threat' condition) and half were assigned to receive 'coping' messages describing the rationale and effectiveness of hand-washing for reducing the risk of infection. This study provided encouraging confirmation that our intervention development was proceeding correctly. Our measures of intentions proved sensitive to group differences, and the behavioral determinants we included in the study explained a substantial proportion of the variance in intentions. The study also provided useful indications that our high threat message might increase hand-washing intentions, that providing hand-gel might be beneficial, and that it would be necessary to actively manage the risk of selective dropout in the intervention group.

Behavior change techniques used in the website

See below for a list of the theory-based behavior change techniques employed in the intervention; these are described and numbered corresponding to a standardized, published taxonomy [5]. The first session comprised 10 core pages, and each subsequent session contained 3-4 core pages, that were viewed by all users. In sessions 2, 3 and 4 additional pages were then shown, tailored on the basis of users' scores on three items assessing their current hand-washing frequency, belief that hand-washing would prevent virus transmission and perceived difficulty of carrying out the behavior (the two strongest theory-based predictors identified in pilot work, [3]. The core pages were 'tunnelled' to ensure users received essential intervention components [6,7]; after viewing these pages, all users were encouraged to navigate freely from a menu with additional information and advice.

Establishing necessity of behavior

1. *Providing information about behavior/health link*[1]*.* Information in Session 1 and tailored information in further sessions explained the link between virus transmission and hand to mouth, eye and nose contact.
2. *Providing information on consequences*[2]*.* In session 1, the potential consequences of seasonal colds and flu were given, particularly for those in 'at risk' groups. Specific information on the outbreak of swine flu and the associated risks were included to increase motivation, as this risk information was found during piloting to increase intentions to follow hygiene advice [4]. Users also viewed a research example of the positive impact of behavior on illness in a community (the US Navy).

Increasing self-efficacy for behavior

3. *Providing instruction*[8]*.* Detailed instructions on the correct hand-washing procedure, using both soap and water and anti-bacterial hand gel, were included in the core messages; this advice was derived from the United Kingdom National Health Service guidance.
4. *Prompting barrier identification*[5]*.* Barriers that were addressed were identified during piloting [3]. Core messages in each session focused on dispelling common beliefs that may act as barriers such as the belief that catching a cold strengthens the immune system. Users with low self-efficacy were then given tailored advice on overcoming barriers such as not having enough time, being unable to access hand-washing facilities, or coping with side effects such as dry hands. Further information on using hand gel to facilitate hand hygiene was available in the menu section.
5. *Time management*[26]*.* Using antibacterial hand gels was promoted throughout the website in both core and tailored messages as a quicker method than soap and water. Tailored pages proposed daily routines with hand-washing included.

Increasing frequency of behavior

6. *Teaching to use prompts or cues*[15]*.* In session 1 users were provided with specific cues for hand-washing (e.g. when returning home, before eating, and after using the toilet). Tailored messages in later sessions included situational cues outside of the home (e.g. after shopping), and transmission cues (e.g. after being with someone with a cold or flu).
7. *Intention formation*[4]*.* Users were shown core messages reinforcing hand-washing frequency. For low frequency hand-washers, tailored messages included the medical team's advice on the behavior and effective frequency.
8. *Prompting practice*[17]*, goal setting*[10] *and agreeing on a behavioral contract*[16]. In session 1, users completed a hand-washing plan. Feedback was provided with the option to review and improve the plan where necessary. Users were then encouraged to print, sign and post up the plan.
9. *Prompting review of behavioral goals*[11]*.* Low frequency hand-washers received feedback in the last two sessions encouraging them to review and re-commit to their hand-washing plan within the menu section. This section was available to all users.

Providing support for behavior

10. *Providing general encouragement,[6] contingent rewards[14] and feedback on performance[13].* Tailored positive feedback was given to all users in the final two sessions encouraging further progress and/or acknowledging success.
11. *Providing information about others' approval[3].* Core messages referred to the medical team's approval of high levels of hand-washing, and identified social approval for good hygiene practices.
12. *Prompting identification as a role model[21]*. In session 4, core pages promoted positive associations with frequent hand-washing. Tailored messages for users with high levels of hand-washing encouraged them to support their household to increase the behavior too.
13. *Relapse prevention[23].* The menu section in sessions 2, 3 and 4 provided encouragement and relapse prevention advice for users who had contracted a viral infection.

In addition to the techniques included in the taxonomy [5], session 2 included a core message intended to induce mild 'disgust' towards dirty hands in order to motivate hand-washing [8]. The page showed a photograph of a dirty hand and suggested that hands could be covered with 'dirty' viruses that were not visible.

References

1. Morrison L, Yardley L. What infection control measures will people carry out to reduce transmission of pandemic influenza? A focus group study. BMC Public Health 2009 Jul;9:258. PMID:PMC2720966
2. Green LW, Kreuter MW. Health Promotion Planning: An Educational and Ecological Approach. Mountain View, CA: Mayfield; 1999. ISBN:0767405242
3. Yardley L, Miller S, Teasdale E, Little P. Using mixed methods to design a web-based behavioural intervention to reduce transmission of colds and flu. J Health Psychol 2011 Mar;16(2):353-364. PMID:20929941
4. Miller S, Yardley L, Little P. Development of an intervention to reduce transmission of respiratory infections and pandemic flu: Measuring and predicting hand-washing intentions. Psychol Health Med 2011 May:1-23. PMID:21644184
5. Abraham C, Michie S. A taxonomy of behavior change techniques used in interventions. Health Psychol 2008 May;27(3):379-387. PMID:18624603
6. Danaher BG, Boles SM, Akers L, Gordon JS, Severson HH. Defining participant exposure measures in Web-based health behavior change programs. J Med Internet Res 2006 Aug;8(3):e15. PMID:16954125
7. Danaher BG, McKay HG, Seeley JR. The information architecture of behavior change websites. J Med Internet Res 2005 May;7(2):e12. PMID:15914459
8. Curtis VA, Danquah LO, Aunger RV. Planned, motivated and habitual hygiene behaviour: an eleven country review. Health Edu Res 2009 Aug;24(4):655-673. PMID:19286894
