# Supplementary material for: Evaluation of a Web-Based Intervention to Promote Hand Hygiene: Exploratory Randomized Controlled Trial
Source: J Med Internet Res. 2011 Dec 9;13(4):e107. doi: 10.2196/jmir.1963 (PMC3278093; doi:10.2196/jmir.1963)
Supplement: Supplementary file 3 [file jmir_v13i4e107_app3.pdf]

UNIVERSITY OF
Southampton
School of Medicine

PRIMIT: A Primary Care Trial of a Website Based Infection Control Intervention – Pilot Study

PATIENT INFORMATION LEAFLET MReC number: 08/H0502/14 (version: 4, 3/7/09)

We would like to invite you to take part in research to test an interactive website that we are developing to help prevent people catching and passing on respiratory infections (flu, coughs, colds, sore throats). To help you decide if you would like to take part, please read through the information below.

What is this research for?

The research aims to develop and test a website that has information on ways to lower the chances of catching and passing on cold and flu viruses in people's homes. This is important because these infections affect many people every winter, and as well as being unpleasant, they can cause time off work and interference in normal life. They can also be dangerous in households where people get lots of infections, are at risk, or have other health problems.

This study is also important because of concerns about 'pandemic flu', such as Swine Flu. A pandemic is where a new flu virus causes more sickness than normal, and more people of all ages become unwell. It is likely that a pandemic flu virus is caught in the same way as other viruses, so these methods of reducing the risk of infection should also help us during the current pandemic.

Who can take part?

Your name was chosen randomly from your doctor's list to make sure that the people taking part are typical of the practice. We are looking for people:

- **Aged 18 or over**
- **With access to the internet and an email address**
- **Living with at least one other person (adult or child), who will allow you to pass on information to us about their cold or flu-like illnesses**

You do not have to suffer from colds and flu, or be at risk from them, to take part in this study.

What will happen to me if I take part, and what do I have to do?

The aim of the study is to design and test a website that shows people how to avoid catching infections and passing them on within the household. For this part of the study, the website has been designed and we need to test it to make sure that it is helpful. We are asking you to consider taking part in this pilot stage.

Everyone in the study will have access to the website, but to see if it works two-thirds of the participants (the intervention group) will have access immediately, and one-third after 4 months (the control group).

Whether you are in the intervention or the control group will be decided randomly, which is the best way scientifically to show whether the website works.

If you are in the intervention group you will be sent emails across a 3 week period asking you to log on to 4 different website sessions, read the information, and answer some questions. You will also be asked to do so again after a 2 month break. If you are in the control group you will be sent an email asking you to answer some questions online, and then to do the same again 3 months later. People in the control group are just as important as those in the intervention group. If you are allocated to the control group it is important that before you get access to the website that you just go on as normal without trying to do anything different.

What else does the taking part in the pilot study involve?

Making a note of infections for you and your household.

So that we can track how infections spread, you would be asked to fill out a brief online questionnaire each month for 3 months about cold and flu-like illnesses in your household, which will take 1-2 minutes. You will also be given post-cards to return if you need to see the GP for infections, or if you need hospital admission.

The questionnaire asks you to fill out information for yourself, and on behalf of at least 1 other member of your household.

If they are ill with a cold or flu-like illness, you will be asked to give:

- their age,
- what type of illness they had,
- how long they had it for.

You will need to ask the people you live with if they are happy for you to pass this information on to us. They will not need to do anything else, or have any other involvement with the study. **You do not need everyone you live with to agree to pass on this information, but if NO ONE in your household will allow you to pass on this information to us, you will not be able to take part in this study**.

The information you collect will be anonymous and we will never know the names or any other medical information of the other household members. All the information you give us about your infections and the infections of people in your household will be on secure computer systems, and will not contain anything which may identify you or them.

Will any other information be collected?

With your permission, your GP notes will be accessed to find out about relevant previous infections and treatments, and any other treatment or care you might have needed for this infection. All these details will be kept fully confidential and used for research only. We do **not** need to access the medical notes for other people in your household.

Is there anything else involved in taking part in the pilot study?

No, however there are some additional **optional** studies which we will contact you about in the future if you are interested. If you would like to know more about these studies please tick the box on the consent form.

What are the advantages and disadvantages of the study for me?

The main advantage is that your help with this study will give us important information that could help lots of people catch less colds and flu in the future. It will also help doctors and nurses better manage patients. The only disadvantage in taking part is the time taken.

What will happen to the results?

The research will be published in medical journals. We will provide you with a summary of the results of the survey, but final results of this study will not be available for 3-5 years.

Do I have to take part, and does this study affect my rights?

Taking part in the study is voluntary. It is up to you to decide. If you decide to take part, you are free to withdraw from the study at any time without giving any reason and without affecting your current or future treatment in any way. Taking part in this study does not affect the care your doctor/nurse gives you, or alter your rights to compensation or right to complain under normal NHS procedures.

Will the information I give be confidential?

All the information will be kept fully confidential. Even your GP will not see your answers to the survey. Your name will not appear on any papers or reports. To keep your information confidential all questionnaires will be identified by a number only, and stored on password protected computers in locked buildings which are alarmed when staff are not there. The computer based systems have secure encryption to ensure confidentiality for any data collected or sent over the internet.

What if something goes wrong?

If you have complaints about the way your illness was managed, this study will not affect your normal rights to pursue a complaint within the NHS in the normal way.

What next?

If you have decided that would like to take part, please:

- **sign the Consent Form to agree to participate**
- **post the top 2 copies of the Consent Form back to the research team in the enclosed Freepost envelope.** You need to keep the bottom copy of the consent form as a record for yourself.

You will then be emailed with the link to the survey.

If you would like more information or you have any questions you are welcome to:

- tick the box on the Participation Form to show that you would like a member of the research team to contact you and return it in the Freepost envelope provided.
- attend group sessions run in the practice explaining more about the study. Please contact the Research Assistant as shown below for more details.
- contact the Research Assistant Sascha Miller, 3063 Shackleton Building, University of Southampton, Highfield Campus, Southampton, SO17 1BJ (Email: sm2t07@Soton.ac.uk or Tel: 023 8059 7222).
  (N.B. this number is only for queries regarding the study; if you have an urgent medical problem please contact your doctor in the normal way).

If you would like more general information about medical research you can also contact your local primary care trust (023 8087 4545).

Who is organising, reviewing and funding the research? The study is funded and reviewed by the Medical Research Council, coordinated in two sites in England. Southampton University is coordinating the study locally. It has been approved by the Multi-Centre Research Ethics Committee (application no.08/H0502/14).

Thank you for taking the time to read this Patient Information Leaflet and considering participating.
