# Supplementary material for: Evaluation of a Web-Based Intervention to Promote Hand Hygiene: Exploratory Randomized Controlled Trial
Source: J Med Internet Res. 2011 Dec 9;13(4):e107. doi: 10.2196/jmir.1963 (PMC3278093; doi:10.2196/jmir.1963)
Supplement: Supplementary file 4 [file jmir_v13i4e107_app4.pdf]

Protocol (version 4 3/7/2009)

1. Title: A PRImary care trial of a website based Infection control intervention to Modify Influenza-like illness and respiratory infection Transmission (1.2 Acronym PRIMIT trial.)

2. 1 What is the problem to be addressed. 2.11 Winter respiratory tract infections (RTIs) and influenza. Influenza and other respiratory viruses result in overstretched primary care services, and also hospital bed shortages due to the cardiovascular and respiratory complications, especially when viruses causing influenza like illness (ILI) circulate in the community[1 2-5 6-8]. Minimising the spread of respiratory infections could have considerable benefit for both patients and the health service.

2.12 Pandemic flu. The main reservoir of influenza virus is the bird population. Pandemic flu occurs when a new virulent strain develops which is transmissible between humans, and where the human population has little or no prior immunity. The last pandemic was in 1968, and a pandemic is expected - the longest inter-pandemic gap previously was 39 years. Given the capacity of the highly pathogenic H5N1 virus to adapt and change, the next pandemic could be a transmissible virus developed from the H5N1 virus, and if the morbidity and mortality pattern are similar (currently mortality 40%), then the impact on health and society could be potentially significantly worse than the 1918 pandemic. Interventions to minimise spread of influenza could potentially help modify the pandemic curve and help maintain the viability of health and other public services. There has been widespread concern in the public and the media, increased interest in the uptake of influenza vaccine, and expressed anxiety and helplessness (http://news.bbc.co.uk/1/ hi/health/4341496.stm). Although providing advice on personal protective measures to take could provide an important means of relieving public anxiety[9], research on compliance with protective measures during the SARS outbreak indicates that scepticism about protective measures is common and advice will only be seen as credible - and therefore effective in changing behaviour and relieving anxiety - if it is evidence-based[10].

2.13. Making the diagnosis in ILI: spectrum bias, population behaviour. A systematic review identified the three-fold combination of the presence of fever, cough and acute onset to be the most predictive clinical features of laboratory diagnosed influenza when influenza virus is circulating in the community.[11] The probability of influenza infection also increases with increasing fever[12]. The assumption is generally made that during a pandemic the positive predictive value (PPV) of a clinical diagnosis will increase; however one of the major unknowns is estimating what is likely to happen to the PPV when people who would not normally contact the doctor then do contact the doctor - as is likely to happen with widespread anxiety in a pandemic situation. This would be expected to reduce the PPV, as has been explicitly acknowledged in the Department of Health guidelines for the management of pandemic flu. It may be that by using 'harder' clinical signs (such as the level of fever) the predictive value can be maintained even when such 'spectrum' changes occur due to the behaviour of the public. Equally if patients contact health providers very early in the illness as patients are being encouraged to, and is in any case likely in a pandemic (e.g. the first 12-24 hours compared to 24-48 hours) the predictive values of symptoms may change. Since in this trial we will be documenting what viruses are circulating, this information will also allow us to perform a nested diagnostic study to estimate the PPV (crucial for the targeting of treatment) and also the NPV (crucial for reassuring patients that they do not have flu if they are being denied treatment) according to such potential 'spectrum' changes. This information with be useful both in inter-pandemic years as public behaviour changes in response to heightened anxiety about influenza, and also in a pandemic.

2.14 Limiting transmission of influenza and other respiratory infections. Infection control and the behaviours related to infection control are one of the priorities in the influenza research call by the MRC. The policy of providing anti-virals early (within 48 hours) to patients with ILI in a pandemic is already in place as part of the DOH clinical guidelines. What is less clear is whether other infection control measures will work**.** Community based major public health interventions are unlikely to be possible interventions for a trial in a non pandemic situation (e.g. it is unlikely patients would be prepared to wear masks in public or modify travel/shopping/leisure arrangements, school closure is not possible etc). Similarly, intensive behavioural interventions (e.g. multiple counselling session) would not be feasible in any pandemic. However, a simple low technology home/family based intervention that could be used in a pandemic is feasible, but could also be assessed - and prove useful – in non pandemic years.

Method of transmission and infection control. Influenza is predominantly transmitted by large droplets (particularly within three feet of the infected person), by touching contaminated surfaces (where the virus can be viable for some time) then touching nose mouth or eyes, and by fomites. These patterns of transmission are common to many other respiratory viruses, although viruses vary somewhat in the importance of the different routes of transmission. The use of masks, handwashing, washing down surfaces and cough hygiene have all been recommended in health care settings and the community[13-16]. For masks there is little specific information for influenza; some evidence from the SARS outbreak suggested a dose response relationship between mask wearing and transmission, but the retrospective design makes firm conclusions difficult [16]. Cough hygiene in the index case (covering mouth when coughing, using disposable tissues) has been recommended, as is staying away from the patient by at least 3 feet - given the evidence of greatest risk being in the immediate proximity of the

case[14;15], but there again there have not been adequate trials to test this approach. Although there are issues of generaliseability, handwashing with soap or with an alcohol cleanser has the best evidence and is likely to limit transmission of respiratory infections in both adults and children[1617;18 19 20]: most controlled studies identified have been positive [16] (see section 2.4) and a review of case-control studies during the SARS outbreak also suggested handwashing may have been protective [21]. Other measures which may be effective include washing down surfaces using a household disinfectant, [14 15]the use of a separate bedroom/living area where feasible, and separate towels.

Why this study? This study addresses key issues in the clinical research agenda (infection control; diagnosis) and provides methodological interest in three areas: a) using a clear theoretical framework to develop a low cost novel intervention (a web-based intervention - which is rare in primary care) b) using novel, predominantly postal recruitment methods, for large patient cohorts c) assess spectrum bias in the diagnosis of community infections.

2.2 What are the principal research questions to be addressed?

1) To develop, then estimate the effectiveness and cost-effectiveness, of a website-based, theoretically-derived, infection control intervention

2) To estimate the positive and negative predictive values of clinical symptoms and signs for laboratory confirmed influenza according to different spectrums of disease defined by consultation behaviour and prior illness duration

2.3 Why is a trial needed now? Since current advice on infection control is based on relatively limited trial evidence, a trial of the effectiveness of personal infection control is necessary and timely. There is little understanding of the attitudes, beliefs and anxieties of the public regarding influenza transmission and infection control, and little evidence about how the impact of diagnostic spectrum bias might be minimised. This is particularly important given high levels of public concern regarding influenza, and its prevention. In a short time frame (18 months) this study will develop a low cost technology (an interactive website), which will use a theory-based tailored approach to advice, and which could rapidly be made available should a pandemic strike. Following the development phase we propose trialling the effect of the website. This study will also provide very useful outputs for non pandemic years: it will provide evidence about the effectiveness and cost-effectiveness of a simple and inexpensive intervention for respiratory tract infections and influenza - which are the commonest illnesses in the population - and inform the issues surrounding spectrum bias in the diagnosis of flu-like-illness.

2.4 Relevant systematic reviews. We have searched the Cochrane data base, Medline, Embase, Psyclit, and also Google Scholar, using the terms respiratory tract infection, masks, infection control, handwashing, booklets, pamphlets, and influenza which we have reviewed above (Section 2.14). Regarding evidence specific for the transmission of RTIs the WHO writing group have reviewed the existing controlled studies for hand washing: of the 9 studies comparing intervention with a control (one was a historical control) 8 out of the 9 studies reported positive results (ranging from a 10%-50% reduction in infections). However, there are generaliseability issues regarding the setting of interventions (health system; social context; site of intervention), and little research specific to influenza nor for other respiratory infections managed in families. We have also performed a broader Cochrane systematic review of the literature on the use of information (booklets, leaflets, and interactive websites/computer programmes) for the management of respiratory infections and other normally self limiting illness (as part of an MRC studentship for P. Andreou). Our review found that 4 out of 6 studies that simply mailed the intervention by post found significant differences in consultation behaviour; the only study that used a health diary similar to the one proposed in our study reported a 15% absolute difference in self treatment behaviours with a mailed booklet (36% vs 51%). The pamphlet we developed for predominantly respiratory illness – which had no clear theoretical framework - was nevertheless surprisingly effective in modifying consultation behaviour for respiratory infections (OR 0.83, NNT 25)[22], and even more effective for those that had consulted previously for respiratory infections. We have also achieved similar changes in behaviours with other booklets[23-25]; and have recently developed successful structured theoretically based booklets for exercise[26] and for self-management of attacks of vertigo[27;28]. There is some evidence that the effectiveness of previous infection control interventions in pandemic and non-pandemic contexts may have been limited by variable levels of compliance[10;29;30]. While compliance rises with anxiety during a pandemic [31], it can also be increased by interventions that use psychological theory to identify and address the beliefs, concerns and behavioural practices that influence adherence behaviour [32 33 34 35]. To date we have found no booklets nor website for respiratory infections that have had theoretical development. Rigorously developed information provision for infection control in the form of an interactive website, backed up by regular automated email prompts, addressing an illness that has high public interest, and targeted to allow for individualised advice should be rather more effective in non pandemic years than our previous booklets; furthermore such an intervention is likely to be still more effective for a pandemic.

2.5 How will the proposed trial differ from of complement planned, ongoing or recently completed trials?
We are not aware of any interactive websites for control of respiratory infections, and no ongoing trials.

2.6 How will the results of this trial be used? This study will provide:

In phase I (development and piloting) : a) Information regarding the attitudes, beliefs and anxieties regarding influenza and its transmission both for pandemic and inter-pandemic years; b) a low cost simple technology (an

interactive website) which could reduce public anxiety and improve patients' attitudes, intentions and enablement regarding managing and preventing influenza and other respiratory infections within families; the intervention will be made available immediately should a pandemic occur; even with modest effectiveness the intervention is likely to be highly cost-effective, and rapidly and widely available to most of the UK population;

In Phase II (the main trial) . This study will: a) assess whether a simple strategy to control infections in families is likely to be effective in non-pandemic years - the intervention will be available at the end of the trial for the whole UK population; such an intervention could potentially make an important difference if and when a pandemic influenza does occur, as compliance is likely to be higher in a pandemic situation**.** b) A virological sub-study will provide important information about the pattern of viruses circulating during the trial period to inform generalisability of the intervention; it will also inform early diagnosis and treatment of flu-like-illness; assess the likely magnitude of diagnostic spectrum effects if the consultation behaviour of the public changes; and provide some information about how robust individual symptoms and signs are to spectrum bias.

2.7 Safety of patients. There are no safety issues in this trial

3.1 Proposed trial.

Development phase (12-18 months). We will a) perform qualitative and survey work to determine the key concerns, beliefs and attitudes related to influenza, pandemic influenza, and infection control behaviour

Trial phase: Pilot trial. We will first pilot the intervention and outcome measures.

Main trial:

Adult patients (aged 18+) living with at least one other person. Patients whose GP notes show a skin problem that may affect handwashing ability, and those who live in households where no one else is willing to provide illness feedback will be excluded. Participants will be randomised to:.

1) Access to an interactive website by a personal password, providing tailored advice; this will be reinforced by email prompts and reminders to use the website; patients will be advised to use hand-washing and a range of infection control measures both within and outside the family
2) Normal care (access to the website at the end of the trial)

Outcomes: transmission of ILI and other infections between family members, severity and duration of ILI and other respiratory infections.

Virological sub study: based on laboratory confirmed virological diagnosis, we will document the nature of circulating viruses, estimate whether transmission differs according to the type of viral illness; provide estimates of the predictive value of clinical symptoms and signs according to the spectrum of illness (defined by contact with the NHS and prior duration of illness).

3.2 What is the proposed trial design. An RCT, with a development phase and a nested viral study.

3.3 What are the planned trial interventions.

Infection control website. Our key intervention will be access with a personal password to a website providing advice on infection control behaviours, with reinforcement by email reminders to use the site. A website is likely to be the most efficient and cost-effective mode of delivery in a pandemic as the majority of homes now have access to the web and the figure rises each year (National Statistics; August 2006); currently more than 60% of households have access to the web and the figure goes up year on year by 5% each year in a linear fashion - which suggests that even by the end of this trial 80-90% of families could have home access to the web. A recent US study confirmed that most people would use Google as their first source of information in a pandemic [9]. A website will facilitate tailoring of the intervention to the individual, and personalised action planning **-** and thus is the most likely to be effective[36;37 38;39 40;41]. Having both personalised advice via the web and behavioural prompts (regular email contacts) will provide mutual reinforcement of consistent messages[25 23]. The website will be tailored to provide targeted advice based on the factors that have been shown to be important influences on behaviour in the qualitative study and survey carried out in the development phase. These are likely to include: family circumstances and lifestyle factors that may affect which behaviours are feasible and preferred (e.g. availability of suitable space to isolate infected family members, skincare problems); beliefs and concerns regarding infection transmission and control; strategies for overcoming barriers to adherence (e.g. forgetting, negotiation with other family members); current infection status of family and adherence levels (i.e. specific advice will be given when the individual or another member of the family is ill, and depending on the current level of reported adherence to each behaviour). The randomised individual (the index person) will be the main target for behaviour change which should limit spread from that person to members of the household and vice versa. However the maximum effect on illness in the family will be if all members of the household participate and change behaviour; this will be encouraged at invitation, and all members of the household will be encouraged to use the website. The extent to which is it possible to engage other members of the household, and the optimum methods for achieving this will be explored in the development phase. During the pilot, participants will be made aware that they will not be able to participate if all other members of their household refuse to provide additional information on their cold and flu illnesses. Through the website and the individual passwords

we will be able to monitor whether patients have used the website, and how often. Patients who have not used the website will be prompted by email to use the site. Following accessing of the website we will generate regular automated emails at monthly intervals as a reminder to use the website; these reminders will enable us to monitor compliance in the intervention group and give targeted advice. We will provide further emails prompts to all patients when influenza virus has started circulating in the community. During the development and pilot phase we will examine the acceptability and feasibility of using the website as the main method of interacting with patients for the whole trial process (including outcome data collection), with paper-based reminders only when web-based measures are not completed. In the development phase we will also explore the need to provide supplies of some materials to help in infection control (a handgel dispenser sufficient to cover periods of respiratory illness).

Target behaviours. The intervention will encourage the use of regular handwashing throughout the winter months using normal soap or alcohol based handgel both within the family and in other settings. Then when somebody in the household has developed an ILI or other RTI (defined below), the aim is to influence the following behaviours: 'hygiene' in the index case and among family members- handwashing with soap or alcohol gel before any food eaten and after any skin or clothes contact with index case; three times daily washing of kitchen work/dining surfaces using a normal household cleaner; covering mouth when coughing (also see mask wearing, but mask wearing in non-pandemic years may not be acceptable or possible); minimising skin contact with family members, using disposable tissues, using separate towels and physical distancing where possible (more than 3 ft distance; minimising time in the same room; living/sleeping in separate rooms/bedrooms where possible). Wearing masks by the patient and family when in the same room as those with influenza (using FFP2, FFP3,N95 or equivalent masks) may limit the spread of large droplets by coughing; but it is the least likely behaviour to be acceptable in inter-pandemic years; our qualitative study will indicate how acceptable mask wearing is likely to be and inform whether masks are provided in the main trial. Based on the literature to date the key behaviour which has the most evidence is handwashing, and so this will be emphasised, but the website will help patients to develop individualised action plans based on their circumstances. Although a development phase cannot realistically determine with any power the effectiveness of each component of behaviour change in modifying transmission, in the analysis of the main trial we will be able to explore this. A development phase will nevertheless be able to provide some insight into the effectiveness of the different behaviours; development is also necessary for a complex intervention to allow us to understand and model the key factors determining the likely implementation of these behaviours (http://www.mrc.ac.uk/pdf-mrc_cpr.pdf):

Theoretical basis. The behavioural literature and empirical trial data suggest that a tailoring approach that personalises advice to the individual is likely to be most effective in changing attitudes and behaviours [37 38;39;41]. Psychological theory will be used to identify the factors on which advice must be tailored. The Theory of Planned Behaviour will be used as the primary basis for developing the tailored intervention, as there is evidence that components of the model that are amenable to change by intervention are key predictors of health-related behaviour[33;35 26]; a meta-analysis of 47 studies also shows that these attitudes/beliefs can be experimentally manipulated and do then change behaviour[42]. We have experience of several successful studies which have used structured booklets based on the Theory of Planned Behaviour combined with traditional behavioural techniques [26-28;28]. The Theory of Planned Behaviour identifies the proximal factors influencing behaviour (perceived costs/benefits, others' attitudes, barriers/support). To investigate additional factors that may directly or indirectly influence behaviour it can be useful to draw on more than one theory; for example, the Theory of Planned Behaviour can be usefully supplemented by Self-Regulation Theory[43] and by considering volitional factors influencing the implementation of intentions [44]. We will therefore employ a causal modelling approach[34;45] to identify additional relevant influences on behaviour and appropriate corresponding methods of intervening to change behaviour. We will use Protection Motivation Theory [46;47 48] to look at perceived threat of infection (personal vulnerability, consequences, and fear), since mask-wearing in the SARS epidemic was related to perceived susceptibility to infection as well as perceived benefits of mask-wearing [49]. We will use the Self-Regulatory Model[50] to examine personal models of viral infection (e.g. perceived cause, nature and timeline of infection) that may affect attitudes to infection control (e.g. beliefs that respiratory infection is entirely airborn or occurs too quickly to be prevented), as there is limited evidence that these may have influenced behaviour during the SARS epidemic [31;51]. We are aware of no theoretical work on factors influencing our target behaviours in the context of nonpandemic respiratory infection. However, based on the literature on behaviour to limit STD transmission we will assume that infection control will require negotiation between the patient and other family members[52]. We will also use evidence-based methods of assisting patients to carry out their intentions regarding preventive behaviour [53] including: a) planning and cueing of behaviour, for example assisting the individual to set their own behavioural 'targets' for different infection control behaviours, and to make an explicit commitment to these targets (a signed list and website record), where possible in collaboration with members of their family; b) identifying situations when lapses may occur, and suggesting coping strategies; c) using a paper or web-based diary to self-monitor the target behaviours during episodes of RTI (this will automatically trigger reinforcement and advice relevant to self-reported behaviour) [;54,55;56 57-59]

Developing and modelling the intervention:

- **0-6 months, qualitative work and development of website content.**. Before a maximally effective website can be developed we need to better understand patients' perspectives. In depth semi-structured interviews with up to 30 patients will explore attitudes to infection and infection control behaviours, likely behaviour in a pandemic, as well as feasibility and process issues for the trial. We will initially use a maximum variety sample (age <65 and >65, children living in the household, gender, and social class) and perform thematic analysis; time permitting we will extend the study using techniques from grounded theory, including the selection of a theoretical sample. The semi-structured interview guide will elicit beliefs about infection; previous and current experience of hygiene measures; the perceived advantages and disadvantages of carrying out the target behaviours, and factors that would hinder or assist carrying them out; perceived social context/support; perceptions of the proposed website and the role of health professionals; and issues relevant to the process and documentation in the trial. To help us to understand the influence of family dynamics we will invite patients to include other family members in the interviews. We will show a draft version of the information we are developing for the website to patients at the end of the interview; based on the analysis of the interviews and patients' comments the information will be modified in an iterative fashion. The qualitative work will continue throughout the development phase and also in the pilot and main trials to understand issues of process and to help explain the quantitative findigns (**Deliverable 1 (8 months): report of qualitative work documenting likely target attitudes and beliefs for key behaviours)**
- **6-12 months: survey and development of the website**. The specific beliefs identified in the qualitative work will be used as the basis for a questionnaire survey of those who took part in the interview study and also 200 randomly selected patients from practices in the locality of the study centre. The purpose of the survey is to quantify the prevalence and strength of these beliefs, and to identify the key attitudes and beliefs discriminating between those who do and do not intend to carry out infection control behaviours, since these are most likely to influence behaviour, and should therefore be the target for provision of tailored advice by the website[35;36] . These attitudes and beliefs will be assessed for inter-pandemic years, but we will also ask the same questions for pandemic years (having provided a description of the likely impact of a pandemic). This will allow us to determine whether different factors may operate in pandemic years (e.g. higher levels of concern for sick family members), and to ensure that the website is designed to also address these factors (e.g. emphasising the need to protect one's own health in order to care for family). From the results of the qualitative work and survey we will have sufficient information to complete programming of the website to carry out the tailoring of the intervention and personalised action planning. One of our team (LY) has already had experience of developing a tailored health promotion interactive website [www.balancetraining.org.uk;[60]]. We will initially present information addressing the most common motivational beliefs, allowing patients to also self-select further information relevant to their particular concerns using a set of 'frequently asked questions' that they can click on for additional information (e.g. after explanation of how respiratory infection can be prevented by handwashing additional questions might include 'how effective is handwashing?', 'how can I avoid getting dry skin?'). Tailoring is then achieved by algorithms linking responses to key questions about the website user's situation (e.g. children, living circumstances) and beliefs/intentions to a library of messages, so that website users automatically receive a unique combination of messages tailored to their particular situation and concerns. For example, only parents will be presented with suggestions for how to encourage children to wash their hands; further personalised motivational information will be presented to those who indicate that they are not yet convinced about the value of carrying out the target behaviours; additional volitional advice to help patients plan and carry out the behaviours will be presented to those who report that they intend to do them but have low adherence for particular behaviours, and this will include advice targeted to overcoming the specific barriers they identify. (**Deliverable 2**: **report on survey work (11 months) confirming key attitudes and beliefs for the behaviours)**

Pilot trial. 12-18 months: piloting and finalising the intervention. Following the completion of the website, we will pilot the trial (i.e. invitation, intervention, and outcome measures) to confirm the extent to which the intervention can change relevant beliefs (instrumental and affective [61], attitudes, subjective norms, perceived behavioural control, intentions and enablement[62], and to confirm the acceptability/feasibility of our intervention and outcome measures. Based on previous tailored website studies [60;63] we calculate that we will need a sample size of 130 per group to have 80% power to test a between-group difference (2-tailed alpha level .05). Preliminary piloting of the web pages has suggested a possible danger of demotivation if patients contract viral illnesses despite getting preventive advice regarding handwashing. To help counteract this and to support patients further we will explore in the pilot phase the impact of adding weekly messages about supporting the immune system (advice to take Echinacea[84] use vitamin C[85] and pelagonium[86], and the use of steam[87]) in half of the intervention group. Supporting the immune system to fight infection provides a different message to avoiding transmission of viruses, and before incorporating this prior to the main trial we need to be sure that the different messages work together. By including immune system messages we will be able to examine the effect of these on hygiene

behaviours. We will also pilot methods of collection of viral samples, and compare the feasibility and acceptability of primarily postal vs. website-based outcome data collection. Further minor modifications to the website and our procedures will be made if necessary based on the findings from this pilot study. (**Deliverable 3: report on piloting (20 months) confirming change in attitudes and intentions for key behaviours.)**

Supply of materials. We are contacting manufacturers of alcohol based hand gels to help supply households with their product for the period of the pilot. We are estimating that it could take up to 8 weeks for some participants to complete the website sessions, and will be asking the companies for sufficient supplies to cover this timeframe. Although they will be able to provide branded products which will be displayed on the website, any branding will be removed on completion of the study. The commercial company will have access to relevant analysis from interviews and the outcome measures of the trial. However, they will not have access to individual participant information, transcripts or interview tapes. The commercial company will not have any ownership of the data collected and will have no rights to publish papers based on the information. A contract will be drawn up outlining the details of the relationship with the commercial company and this will be submitted for NRES inspection.

Control group (normal care). As in the intervention group, the control group will have access to the GP/practice in the normal way for respiratory illnesses and ILI (where low levels of antivirals are normally prescribed). We are not proposing to encourage all patients to access antivirals early in the illness, since the effect would be to reduce transmission rates to substantially below the rates that would be expected in a pandemic (where, even with antivirals, transmission rates are likely to be high, given the low levels of immunity and severity and duration of the illness). In our previous RCTs which have randomised at patient level (including RCTs using information leaflets) there has been little or no contamination of the control groups. In this study, since access will only be possible by a personalised password, contamination is even less likely. Inevitably some patients in the control group will want to go searching on the net for their own information, and to some extent by asking patients to participate we may increase their curiosity about such issues. We will minimise the possibility of this contamination by explicitly raising the issue prior to randomisation, and stressing the importance of a fair test of the website; we will ask patients who intend searching the web not to agree to participation; and we will minimise the incentive to do so by offering access to the website to all control patients at the end of the year. We will monitor any contamination of the control group at the end of the year of follow-up by asking all patients whether they have accessed infection control information by the web and assessing levels of key behaviours of all patients at the end of the trial to determine whether these have changed within the family. Possible contamination of the control group raises the issue of dilution of effect size, but our effect sizes are based on past trials where the sort of level of contamination possible (i.e. availability of access to information) was at least as great as in the proposed study; for example our booklet/leaflet study[22] came at a time where the local PCTs were providing mass mailing of information booklets to many PCTs, and the companies supplying booklets were engaged in active advertising locally.

3.4What are the proposed practical arrangements for allocating patients to trial groups? We propose using a very similar mechanism to our previous leaflet trial[22], which used a novel method to recruit large numbers of patients. 1500 patients from each practice will be identified by the practice staff with the help of research staff. if the practices would like help (the research staff will hold honorary contracts with the PCT). Letters will be sent to patients inviting them to participate. Assuming conservatively at least 66%-70% agree to the main study (we achieved an 80% uptake previously in a similar study and using a questionnaire burden that was higher than the present study)[22] then 1000-1100 patients in each practice should agree. If fewer agree more letters will be sent out in each practice. Patients willing to participate can return the consent form directly to the research centre, or talk with the research team before replying. Those patients wanting more explanation can also attend sessions run by the practice nurse and/or research staff as appropriate. Patients will then have time to think about the study, and can also subsequently contact the research centre later before sending their consent forms back directly to the research centre. Patients can either return the consent from by post (which has previously been the case for the majority[22]), log on to the website to confirm consent (assuming ethics approve this method). If our pilot study indicates that a primarily postal method of trial administration is needed then the trial nurse will then provide lists of patients' numbers and details in each practice to an external randomisation line (the data will including stratification variables: age >65; influenza vaccination status; size of family, children under aged 16 living at home; access to the internet; willingness of other members of the family to use the website; attendance in the previous year with respiratory infections; a skin complaint that may affect handwashing); the randomisation centre will then randomise, stratified by the above variables, and provide the randomisation lists which will be distributed to the practice staff. The practice staff will then send out separate batches of letters to intervention patients (with the personalised website password depending on which intervention they will access) and to control patients (no password). Patients consenting to the diagnostic study will be given instructions on how to take samples, and will see a nurse for demonstration as necessary. Patients will be given the symptom diary for them to complete when an ILI develops. If the pilot study indicates that a primarily website-based based method of trial administration is appropriate then randomisation will be automated and outcome measures collected electronically (with paper-based follow-up of non-respondents).

3.5 Methods of protecting against bias (e.g. masking, blinding)? Blinding of patients to whether they have

received the intervention is impossible. Data entry will be blind to study group. Selection bias and non-response/loss to follow-up bias will be assessed by comparing the socio-demographic/clinical characteristics of those recruited/not recruited and followed up/not followed-up respectively. **Selection bias:** We will document when eligible patients are not entered into the study, the reason why, treatment, and clinical characteristics.[64 65].

3.6 Inclusion criteria. Adult patients (aged 18+) from GPs computerised lists where there is at least another living in the household. **Exclusion criteria.** Patients with severe mental problems (e.g. major uncontrolled depression/schizophrenia;dementia;severe mental impairment - unable to complete outcomes) or terminally ill. Patients who have reported a skin complaints that may affect their ability to wash their hands. Participants who live in a household where no one else is willing to provide information on illnesses via the website.

3.7 What is the proposed duration of treatment (see interventions)

3.9 and 3.10. What are the proposed outcome measures, frequency and duration of follow-up?

- **Primary: Episodes of ILI and RTI, their duration and transmission in families.:** *We hypothesize that the intervention will reduce the number of episodes (by reducing transmission) and hence the number of days with symptoms.* During the feasibility phase we will pilot the use of both paper and web versions of illness diaries, and of monthly questionnaires (both web and paper based) with emailed reminders. Our preference is that index patients will be prompted by email to log onto the website monthly to complete questionnaires about illnesses during the last month since the duration of symptoms can be remembered reliably over a period of a few weeks[25;66]. However those that prefer will be provided with a double sided A4 sheet (and spare sheets in case of families with frequent infections) to provide simple summary diary entries of respiratory tract infection, and will still get the monthly email prompt reminders. For each episode the index person will document: the nature of the infection; the duration of symptoms rated moderately bad (which we have shown is the most likely to be sensitive to change for individuals with cough and fever[25] and can be remembered reliably over a period of a few weeks[66 25]); the number of days where work/normal activities were impaired[25]; whether other family members either had a similar infection before or after the index case, and what the time interval was; whether contact with the health service was needed; and (for the intervention group only) how well the index patient and other family members complied with the target behaviours. We have used diary sheets in our respiratory studies and found good levels of compliance[64;66]. The monthly illness information will be supplemented by a questionnaire at the end of the winter (April) documenting the summary information, and which can be validated against the monthly information. We are currently using a similar end of winter questionnaire in a study of respiratory and ear infections in families (the 'PIPO' study http://www.controlled-trials.com/ISRCTN53286030), and shown it to be feasible and reliable; this questionnaire will particularly provide some information for those individuals not returning their diary or monthly questionnaires. Our experience with questionnaires is supported by illness transmission studies where similar questionnaires/diaries have been used[67]. An illness will be classified based on the records as a potential ILI based on reported fever (or measured temperature >37.5), plus a respiratory symptom (sore throat, cough, coryza) plus a systemic symptom (prostration, headache, severe fatigue, severe muscle aches, severe malaise) i.e. using an analagous definition to the diagnostic sub-study. We will base our definition of RTI on consensus definitions developed in previous studies [30;67] - which have defined a respiratory tract infection as 2 symptoms of an RTI for at least 1 day or 1 symptom for 2 consecutive days. Evidence of transmission will be defined as a respiratory illness developing within a week of another family member (viral shedding occurs mostly in the 5 days after symptoms commence and the average incubation period is 2 days). We will assess the implications of using different cut-offs in defining both symptomatic RTIs and the time defining transmission of RTIs.
- **Secondary. *A*ttendance at the GP practice, and use of health service resources: Notes review.** *We hypothesize that the intervention will reduce the number of health service contacts by reducing the number of episodes.* At the end of the study all patients' GP notes will be reviewed to document admission to hospital for respiratory or cardiovascular complications, whether patients attended the GP for their ILI or other respiratory tract infections[68], whether antibiotics or antivirals were prescribed, and/or any subsequent referrals. Assessment of the notes will be made blind to group, and has been shown to be reliable and unbiased [64].
- **Attitudes and adherence to infection control behaviours.** *We hypothesize that the intervention will change attitudes, beliefs, intentions, and behaviour.* We will assess attitudes, beliefs, intentions, and self-reported adherence for each behaviour on 7 point Likert scales[69] both in the pilot study (after 1 month and 3 months), and in the main trial at the end of the winter (we will randomly choose 1:20 patients for this outcome). These outcomes will be measured in both groups in the main trial only at the end of the winter, to minimise any effect on behaviour change of administering questionnaires in the control group. These outcomes will allow us to check whether the intervention is successful in effecting change and whether outcomes of the intervention are mediated by changes in these variables.To accompany the winter illness questionnaire we will use an adherence questionnaire (the 'Problematic Experiences of Therapy Scale'; PETS) that we have

used in previous studies, including the MRC ATEAM trial, and two vertigo trials[27 28]. To reduce social desirability effects on reporting of adherence, the PETS asks patients to what extent they have been prevented from carrying out the intervention by socially acceptable reasons (e.g. symptoms too severe or aggravated by the intervention; doubts about efficacy; uncertainty about how to carry out the intervention; practical problems such as lack of time or opportunity, forgetting). They are only then asked how often and over how many weeks/months they adhered to each aspect of the intervention. To compare the effects of the intervention as a whole with the effects of the website and the target behaviours, in addition to the main intention-to-treat analysis we will carry out per protocol analyses to determine the effectiveness of the intervention in a) those who logged on to the website and b) those who reported adhering to each of the different target behaviours. We will also examine whether the website was equally effective for all sections of the target population (based on age, sex, family factors, deprivation). For the purpose of these analyses, we will supplement and validate the PETS scores using the prospective records from the web-based diary of compliance to the behaviours in each episode of family respiratory illness (completed by the intervention group only).

- **Socio-demographic and comorbidity data.** We will collect age, gender, social indices based on post code (to calculate the index of material deprivation), co-morbidities from the notes where available, and educational level from the patient. This data will document the generaliseability of the sample, the similarity of trial groups, and explore effect modification due to deprivation or other demographic variables (although we did not find effect modification previously[22]). The issue of deprivation is relevant since currently 25% of those without web access cite costs of equipment or cost of access as a contributory factor to limiting access. We will document how deprivation (measured by the index of material deprivation based on post code) relates both to uptake and effectiveness of the intervention.
- **Diagnostic substudy.** This study will assess the extent to which ILI report by participants is laboratory confirmed influenza (based on pharyngeal samples transported in viral transport medium). This will allow some explanatory information for the trial results (transmission according to the type of viral illness); assessment of the generaliseability of the results; and information to assess diagnostic spectrum bias. There is no widely accepted definition of influenza like illness [70-72 73] but most definitions have a combination of fever plus other respiratory or systemic symptoms. Based on RCGP weekly sentinel practice returns we will inform all patients who have agreed to the virological study to commence this substudy when influenza virus is circulating in the community. This is necessary since we already know that the predictive values will be poor when influenza is not circulating. Patients with fever of >37.5, plus a respiratory symptom (sore throat, cough, coryza) plus a systemic symptom (chills, prostration, headache, severe fatigue, severe muscle aches, severe malaise) will record the range of symptoms and temperature twice daily (using tempadot thermometers[74]) for at least 5 days; the symptoms recorded will be based on previous clinical studies in influenza and respiratory infections[12 11 75 76 12;77]. We have used similar diaries successfully[25;65;66]. Patients will be instructed to take their own viral samples using the simplest method possible: our provisional proposal is to do this by gargling with tap water into a universal container (Dr Carman's lab have recently shown this method to provide valid PCR results compared to conventional naso-pharyngeal swabs and transport media (unpublished data)). The validity of this can be confirmed during the pilot phase, and nasopharygeal swabs will be used as necessary; the feasibility phase will also explore how much written and/or telephone support is needed for patients to take and send their own samples. Virological samples will be posted first class to Dr Carman's laboratory (Glasgow) to facilitate standard analysis using PCR to assess the presence of influenza and other respiratory viruses (Influenza A, B, C; Paraflu 1, 2, 3,4; Coronaviruses; RSV; HUMP (human metapneumovirus); Adenovirus; Bokavirus). Patients will be asked both for the last episode of ILI and the last respiratory infection whether they contacted the doctor (or NHS Direct); and will be asked at the end of the diary whether for this episode they have contacted the doctor (or NHS Direct). The patients participating in this study will also be asked whether they would be prepared to provide similar samples and clinical information should a pandemic occur: having a network of patients in the community already experienced and able to provide clinical forms and samples at short notice could be invaluable in providing information about the clinical presentations during a pandemic; a series of practices with many patients in each area will complement the information generated from the MRC flu watch study (which is collecting information from fewer patients in each practice and over a much wider area).

3.11. Will HSR issues be addressed. We think it likely that the intervention will be both effective, and since it is very inexpensive therefore cost-effective. However, this cannot be assumed – for example it is not impossible that a website might increase anxieties about influenza and increase contact rates. Therefore the resource use incurred and potentially saved (particularly any change in the nature of health service contacts with primary care services) needs to be quantified carefully. We will collect cost and outcome data, to allow an economic evaluation, both disease specific (£/episode and per symptom free day) and generic (£/QALY). The former will be based on the primary outcomes, the latter on QALY values. EuroQol [78] (EQ-5D) - the 5 item EuroQol - will be

filled in at baseline and during an ILI or respiratory infection (at different days to provide an estimate of the profile of EQ-5D for an ILI) for those patients doing the more intensive diagnostic sub-study, to provide a measure of quality of life. Patients who are admitted to hospital will be asked to return a post card informing the study team that they have been admitted and will then be sent another 5 item Euroqol to complete after discharge. EQ5D results will be compared with the other outcomes measured in the study and more disease specific measures of cost effectiveness will be estimated if and as appropriate. Modelling pre-trial in the first three months will be carried out to help inform power calculations for economic outcomes, help the design of data collection, and set up and prepare for the modeling that would be necessary post trial. Regarding the main analysis at the end of the study we will estimate the intervention and follow-up related costs, including the time taken to train staff, surgery attendance, admissions and referrals. Resource use data will be collected by notes review, GP and nurse documentation (e.g. of consultation time) and patient self-report. The use of the Hants/IOW Clinical Data Repository, which included all GP and hospital data, will be piloted to check if it can provide the requisite data electronically. If so, this would be validated against a sample of GP and hospital case notes. We will also explore streamlined computerised methods of collecting data on resource use from GP notes since most practices are likely to be computerised. Although our primary analysis will be from the health service perspective we are also interested in the personal costs. Thus during piloting and in qualitative work we will explore the range of resource use - e.g. over the counter medication, transport to GP surgery – to make sure we are not missing important costs. In the final analysis of the trial, resources will be valued using national rather than local unit costs wherever possible to aid generaliseability of the results. The economic analysis will assess the cost per episode and per symptom day prevented and cost per quality adjusted life year (QALY) from a health service perspective. Sensitivity analysis will explore varying the parameter values of greatest uncertainty. The model will be informed by a detailed review to be done in the feasibility phase of the project of the models prepared for the 3 reviews of influenza antivirals prepared for NICE (http://www.nice.org.uk/).

3.12 What is the proposed sample size?: Transmission of ILI. All calculations are based on the patient randomised (the index person).We assume that our more rigorously developed, interactive, and tailored intervention will have an NNT of at least 25 and an OR of 0.76 - very slightly better than the previous booklets which had none of these features (OR 0.80, NNT 25) [22]. There are other reasons to assume this is a reasonable (if not conservative) effect size: people who have attended with respiratory infections are slightly more likely to be interested in participating, and in this group the effect size from previous study was significantly larger[22]; a 20% reduction in transmission is the effect size seen in most of the studies of handwashing alone[14;15;16 19;20]; a larger effect on behaviour is also likely given the heightened public concern about ILI since the above studies. If 10% suffer ILI (http://www.eurosurveillance.org/em/v10n12/1012-223.asp;http://www.niaid.nih.gov/factsheets/flu.htm [79 80]), the average household size is 2.4 individuals (http://www.statistics.gov.uk) i.e. 24% can be affected by the transmission of ILI either to or from the index patient, and 20% of those with no intervention will develop ILI[81], then 15,074 patients are needed (assuming two-sided significance level of 5% and 80% power), or 16,000 to allow some leeway in assumptions (see table below). **Transmission of RTIs.** 16000 will have greater power to detect transmission of all RTIs, and for continuous outcomes this sample size will be extremely powerful (see table). **Nested diagnostic study.** We assume that: at least 10% of the index trial cohort will report ILI, i.e. approximately 1600; 50% will agree to the virological sub study (n=800) (based on the numbers agreeing to more intensive investigation in a current microbiological study of URTI in our group); flu virus is isolated in at least 50% of individuals presenting with ILI when ILI is circulating [11;82 2]; and that 30-50% of patients are likely to contact the doctor[82] then in the cohort reporting ILI then we should be able to estimate the positive predictive value of a clinical diagnosis in predicting laboratory confirmed influenza with 95% confidence intervals of +/- 10% in both the sample attending the doctor (n=240-400) and the sample not attending (n=400-560). For the analysis of the likely positive and negative predictive values (of symptoms, temperature) at each stage in the illness (12hours,24hours,36 hours etc) we will have greater precision since we can use the whole sample who agree to the diagnostic study.

Range of effect sizes likely for the trial (bold= our target for that outcome)

Effect size	N per group (with complete outcomes where transmission of infection possible either to or from the index person)	Total N required for ILI (assuming 24% where transmission possible to or from index person; and allowing for 20% loss to follow-up)	Total N for all respiratory infections(assuming all cohort whe transmission possible; and allowin 20% loss to follow-up)
**Dichotomous outcomes e..g. transmission (alpha=0.05 beta=0.2)**			
**20% vs 18% (NNT 50;OR 0.88)**	6039	62,906	**15,098**
20% vs 17% (NNT 33;OR 0.82)	2629	27,386	6574
**20% vs 16% (NNT 25 OR 0.76)**	1447	**15,074**	3618
20% vs 15%(NNT 20 OR 0.71)	906	9,438	2266
**Continuous outcomes e.g. days illness (alpha=0.01 (multiple outcomes); beta=0.1) standardised effect sizes**			
0.15	1325	13802	**3314**

**0.2**	746	**7772**	1866
0.3	333	3470	834

* standardised effect size of 0.2 = one day or less of moderately bad symptoms due to ILI or RTI

3.13 What is the planned recruitment rate? We will utilise the English Primary Care Research Network (PCRN) to aid with recruitment and retention. We propose recruiting this cohort over 18 months with 1 year of follow-up; this is based on our previous experience that recruiting using this mechanism 1000-1100 patients per practice should agree in under one year[22]; 18 months will provide sufficient leeway to ensure recruitment occurs. In each practice if fewer than expected agree it will be relatively easy to select and invite more patients.
3.14 Are there likely to be any problems with compliance? We already have good reason to believe that patients are likely to comply, and that a trial of this size will probably find an effect[22;83]; effect sizes should be larger with better development, tailoring and an interactive website.
3.15 What is the rate of loss to follow-up? 20% - based on our previous trials e.g. MRC ATEAM [22;83].
3.16 How many centres will be involved? Two centres and their local Networks (Birmingham, Southampton).
3.17-.18. Data Entry, analysis . Data forms will be scanned from pre printed sheets as in our previous study.[22]
Main trial analysis. The data will be analysed according to the Statistical Analysis Plan which will be approved by the Trial Steering Committee before the commencement of any analysis. One intention to treat analysis will be performed. We will assess the transmission of ILI or other respiratory tract infection - i.e. either between the index case and family members or from family members to the index person - using multiple logistic regression, the duration of symptoms labelled moderately bad days using multiple linear regression, and attendance at the GP surgery using Poisson regression, controlling for stratifiers. We found in our previous trial of leaflets there were no significant clustering effects by practice[22]; our analysis will be based on the index patient to avoid a design effect due to family clustering. For the symptom data the main analysis will be of symptoms in the index person but a secondary analysis will assess symptoms reported for all other family members averaged at the level of the family. Confidence intervals will be reported, and the study will be reported in accordance with the CONSORT statement.
Subgroup analysis (using two sided 1% significance levels). We will assess whether any of the stratification variables are effect modifiers; whether age, deprivation, education level, and co-morbidities predict outcomes or are effect modifiers; whether self reported adherence to each behaviour determines outcome.
Diagnostic study analysis. We will assess the sensitivity specificity likelihood ratios and positive and negative predictive values for symptoms and temperature in the whole sample, and in the sub-samples who would not contact their practice or NHS Direct (either in this or in past episodes). We will use logistic regression to determine which symptoms (and or temperature) individually or in combination are most predictive. We will also estimate the predictive value of symptoms (and or temperature) for laboratory diagnosed influenza at 12 hours after the start of the illness, then at 24 hours, 36 hours, 48 hours, and 48+ hours.
3.20 Has a pilot study been carried out? We have not piloted this precise protocol, but have recruited very similar trial cohorts using similar recruitment mechanisms[22], and have developed similar websites[60]
3.21 NHS cost implications of this trial: based on the cross board clinical trials subgroup's opinion that all components of the study are worthwhile we estimate the NHS costs implications are approximately 600k.
3.22 Over what period is funding requested: 60 months. 0-18 m: recruit and train practices and nurses, qualitative work, development and piloting of website, feasibility study (5-10 practices);18m-36m months recruit patients; 37-48m data collection and follow-up notes review;48 to 54 months: data cleaning, analysis, report writing, presenting study results

4.1. Day to day management? The trial manager and RA will run the study supervised by PL and RH.
4.2-4.4 What will be the responsibilities of the applicants, and of staff working on the grant? Details of trial management: the trial management group (TMG) will meet 6 monthly (more initially) as necessary

- The RAs in each site will be supported by part time secretaries, with help from nurses at each site.
- Prof. Little (PI); expertise in pragmatic RCTs, leaflets/booklets for RTIs, developing clinical decision rules (MRC DESCARTE,HTA UTIS, EU GRACE); lead GP coordinating advice for primary care in DOH pandemic flu guidelines; member of RCGP/BMA influenza pandemic management committee; he will take be responsibility for overall day to day running of the trial
- Prof. Yardley: expertise in health psychology; qualitative methods, and the development of theoretically based information booklets; has already led the development of an interactive website similar to the one proposed for this study, and will lead the development of this website;
- Prof. Richard Hobbs: expertise in large complex primary care based RCTs (MRC BAFTA, MRC CUBE, HTA SAFE) and longitudinal epidemiology studies (HTA ECHOES, BETS), managed across widely distributed multiple practice settings (Director of MidReC and CE-PCRN); will coordinate Birmingham practices;
- Dr Moore: deputy director of the PCRNe South West; experience of recruiting large trial cohorts

(Wessex Vitamin D study 10,000 patients); will coordinate practices in the South and West, and be influential in enabling us to use the PCRN to aid practice recruitment and retention.

- Dr Williamson: expertise in diagnosis/management of RTIs and pragmatic trials; will advise the TMG;
- Mr Mullee (MM) is the named medical statistician who and will supervise the statistical analysis to be performed with PL; MM, with TB and GW, will supervise data management
- Dr Gary Wills: expertise in programming similar websites; will supervise the computing RA;
- Prof. Raftery is a Heath Economist, and will supervise the economic analysis;
- Dr Fleming: expertise in influenza/RTIs; head of the RCGP Birmingham Research Unit
- Dr Bill Carman: expertise in laboratory virological diagnosis; his lab will analyse the viral samples.

FECs are calculated on hours/week, duration, then averaged out (e.g. 10% 9 months= 0.58 hours/week)

4.5 Who is the trial statistician?: Mr Mark Mullee

4.6 Who will be the Sponsor of the trial?: University of Southampton

4.7. Are there any risks/hazards for patients. No hazards are anticipated

4.8 Plans for preparing and documenting clinical data for preservation and sharing? We anticipate a protected period for primary analysis (the time to be negotiated) and data being available following this.

4.9 What is the composition of the TSC? This is to be appointed.

**5.1 Financial summary**	**TOTAL**
Research costs	£1,204,871
TSC/DMEC costs	£1,800
Trial Registration costs (£200)	£200
NHS Treatment costs	£697,140

6.1 Application history. This application falls under the clinical trials and influenza calls. The cross board group considered the outline, supported the main trial and virological study, but asked us to consider:

1) lengthening the recruitment rate and support follow-up; we have added 6 months to the recruitment time; in our previous study we recruited practices and completed follow-up in 2 years for 4000+ patients with a lower level of resourcing per practice recruited and patient recruited ; adding a further 6 months allows for the increased difficulties in the current climate, and we have also increased clerical support modestly to aid with follow-up in the final year (this is now altogether more than 50% more resource available for notes review per patient recruited and over a 50% longer time period than we used in our previous trial[22]); we can add a further 6 months if the Board desires (but that will require another 6 months staff time pro rata)

2) providing more details for the development phase (particularly survey methodology and objectives): we have provided more details of the development phase which will be led by Prof Yardley

3) providing more details of the latest evidence base for the nature and impact of the behavioural change envisaged : we have completed our Cochrane review of the use of patient information in self limiting illness including influenza; we have augmented the discussion of the latest reviews of the evidence base for the nature and impact of the likely behavioural change; the most well documented behaviour is hand-washing which from the limited range of existing studies probably has a slightly greater effect in preventing transmission than our proposed effect size; thus we will concentrate on hand-washing as the primary behaviour of interest; there is less evidence for the other behaviours but all are included as part of both national and international guidance and are plausibly likely to have additional impact to hand-washing

4) setting out clear intermediate milestones/deliverables so that the feasibility and relevance of the RCT could be assessed: we have provided three key deliverables related to the development of the intervention and propose that if the last Key deliverable (i.e. deliverable 3)- i.e. the change in attitudes and intentions based on the pilot study - is not met, then the main RCT does not continue

5) limiting the patients to those with web access; we have modified the protocol to accommodate this; although the number of families with web access is rapidly increasing year on year (last year 60%) there may well still be issues of equity of access to the web based on financial considerations; we will deal with this in both secondary analysis (by estimating whether index of deprivation is related to uptake of the offer of invitation to participate and whether it modifies the effect sizes); we can also explore encourage participation by encouraging web access from local libraries for those families that do not have access to the web at home; finally we will develop a booklet form of the intervention, and although we will not evaluate it separately as part of this trial, it could potentially be made available for families with no access to the web.

Reference List.

(1)http://www.who.int/csr/resources/publications/influenza/WHO_CDS_CSR_GIP_2005_5.pdf . WHO publications 2005.

(2) Zambon M, Stockton J, Clewley J, Fleming DM. Contribution of influenza and respiratory syncytial

virus to community cases of influenza-like illness: an observational study. Lancet 2001; 358 (9291):1382-1383.
(3)MacFarlane J, Holmes W, Gard P, MacFarlane R, Rose D, Weston V et al. Prospective study of the incidence, aetiology and outcome of adult lower respiratory tract illness in the community . Thorax 2001; 56:109-114.
(4) Simpson J, MacFarlane J, Watson J, Woodhead J. A national confidential enquiry into community acquired pneumonia deaths in young adults in England and Wales. Thorax 2001; 55:1040-1045.
(5)Woodhead MA, MacFarlane J, McCraken JS, Rose D, Finch R. Prospective study of the aetiology and outcome of pneumonia in the community. Lancet 1987; 1(8534):671-674.
(6)Meier C, Jick S, Derby L, Vasilakis C, Jick H. Acute respiratory-tract infections and risk of first-time acute myocardial infarction. Lancet 1998; 351:1467-1471.
(7)Madjid M, Naghavi M, Litovsky S, Casscells S. Review: Current PerspectiveInfluenza and Cardiovascular Disease A New Opportunity for Prevention and the Need for Further Studies. Circulation 2003; 108:2730.
(8)Smeeth L, Thomas S, Hall A, Hubbard R, Farrington P, Vallance P. Risk of Myocardial Infarction and Stroke after Acute Infection or Vaccination. N Eng J Med 2004; 351:2611-2618.
(9)Janssen A, Tardif R, Landry S, Warner J. Why tell me now?' the public and healthcare providers weigh in on pandemic influenza messages. Journal of Public Health Management & Practice 2006; 12:388-394.
(10)Cava M, Fay K, Beanlands H, McCay E, Wignall R. Risk perception and compliance with quarantine during the SARS outbreak. Journal of Nursing Scholarship 2005; 37:343-347.
(11)Call S, Vollenweider M, Hornung C, Simel D, McKinney W. Does this patient have influenza? JAMA 2005; 293(8):987-997.
(12)Monto A, Gravenstein S, Elliott M, Schweinle J. Clinical signs and symptoms predicting influenza infection. Arch Int Med 2000; 160:3243-3247.
(13)http://www.who.int/csr/disease/avian_influenza/ guidelines/Guidelines_Clinical%20Management_H5N1_rev.pdf. WHO publications 2006.
(14)Buxton-Bridges C, Kuenhert MJ, Hall C. Transmission of Influenza: Implications for Controlin Health Care Settings. Clinical Infectious Diseases 2003; 37:1094-1101.
(15) CDC. Infection Control Measures for Preventing and Controlling Influenza Transmission in Long-Term Care Facilities. http://www cdc gov/flu/professionals/infectioncontrol/longtermcare htm 2005.
(16)World Health Organization Writing Group. Nonpharmaceutical Interventions for Pandemic Influenza, National and Community Measures. http://www cdc gov/ncidod/EID/vol12no01/05-1371 htm 2006.
(17)Luby S, Agboatwalla M, Feikin D, Painter J, Billhimer W, Atlaf A et al. Effect of handwashing on child health: a randomised controlled trial. Lancet 2005; 366:225-233.
(18)Roberts L, Smith W, Jorm L, Patel M, Douglas R, McGilchrist C. Effect of Infection Control Measures on the Frequency of Upper Respiratory Infection in Child Care: A Randomized, Controlled Trial. Pediatrics 2000; 105:738-742.
(19)White C, Kolble R, Carlson R, Lipson N, Dolan M, Ali Y et al. The effect of hand hygiene on illness rate among students in university residence halls. Am J Infect Control 2003; 31:364-370.
(20)Ryan M, Christian R, Wohlrabe J. Handwashing and respiratory illness among young adults in military training. Am J Prev Med 2001; 21:79-83.
(21)Fung I, Cairncross S. Effectiveness of handwashing in preventing SARS: A review. Tropical Medicine and International Health 2006; 11:1749-1758.
(22)Little P, Somerville J, Williamson I, Warner G, Moore M, Wiles R et al. Randomised controlled trial of self management leaflets and booklets for minor illness provided by post . BMJ 2001; 322:1214-1217.
(23)Little P, Roberts L, Blowers H, Garwood J, Cantrell T, Langridge J et al. Should we give detailed advice and information booklets to patients with back pain? A randomized controlled factorial trial of a self-management booklet and doctor advice to take exercise for back pain. Spine 2001; 26:2065-2072.
(24)Little P, Griffin S, Kelly J, Dickson N, Sadler C. Effect of educational leaflets and questions on knowledge of contraception in women taking the combined oral contraceptive pill: randomised controlled trial. B M J 1998; 316:1948-1952.

(25)Little P, Rumsby K, Kelly J, Watson L, Moore M, Warner G et al. Information leaflet and antibiotic prescribing strategies for acute lower respiratory tract infection: a randomised controlled trial. JAMA 2005; 293:3029-3035.
(26)Little P, Dorward M, Gralton S, Hammerton L, Pillinger J, White P et al. A Randomised Controlled trial of three pragmatic approaches to initiate increased physical activity in sedentary patients with risk factors for cardiovascular disease. BJGP 2004; 54:189-195.
(27)Yardley L, Donovan-Hall M, Smith H, Walsh B, Mullee M, Bronstein A. Effectiveness of nurse-delivered vestibular rehabilitation for chronic dizziness in primary care: randomized controlled trial. Ann Int Med 2004; 141:598-605.
(28)Yardley L, Kirby S. Evaluation of booklet-based self-management of symptoms in Ménière's disease: a randomized controlled trial. Psychosomatic Med 2006; 68:762-769.
(29)Rabie T, Curtis V. Handwashing and risk of respiratory infections: A quantitative systematic review. Tropical Medicine & International Health, 2006; 11:258-267.
(30)Sandora T, Taveras E, Shih M, Resnick E, Lee G, Ross-Degnan R. A randomized, controlled trial of a multifaceted intervention including alcohol-based hand sanitizer and hand-hygiene education to reduce illness transmission in the home. Pediatrics 2005; 116:587-594.
(31)Leung G, Ho L, Chan S, Ho S, Bacon-Shone J, Choy R. Longitudinal assessment of community psychobehavioral responses during and after the 2003 outbreak of severe acute respiratory syndrome in Hong Kong. Clin Inf Dis 2005; 40:1713-1720.
(32)Glasgow R, Klesges L, Dzewaltowski D, Bull S, Estabrooks P. What is needed to improve translation of research into health promotion practice? Ann Behav Med 2004; 7:3-12.
(33)Godin G, Kok G. The theory of planned behavior: a review of its applications to health-related behaviors. Am J Health Promotion 1996; 11:87-98.
(34)Hardeman W, Johnston M, Johnston D, Bonetti D, Wareham N, Kinmonth A. Application of the theory of planned behaviour in behaviour change interventions: A systematic review. Psychology & Health 2002; 17:123-158.
(35)Sutton S. Using social cognition models to develop health behaviour interventions: problems and assumptions. In: Rutter D, Quine L, editors. 1 ed. Buckingham: OUP; 2002. 193-208.
(36)Kreuter M, Farrell D, Olevitch L, Brennan L. Tailoring health messages: customizing communication with computer technology. 1 ed. London: Erlbaum; 2000.
(37)Kreuter M, Strecher V, Glassman B. One size does not fit all: The case for tailoring print materials. Ann Behav Med 1999; 21:276-283.
(38)Lancaster T, Stead L. Self-help interventions for smoking cessation. Cochrane Database of systematic reviews 2006.
(39).Shaw B, Cheater F, Baker R, Gillies C, Hearnshaw H, Flottrop S et al. Tailored interventions to overcome identified barriers to change: effects on professional practice and health care outcomes. Cochrane Database of systematic reviews 2006.
(40)Skinner C, Campbell M, Rimer B, Curry S, Prochaska JO. How effective is tailored print communication? Ann Behav Med 1999; 21:290-298.
(41).Kroeze W, Werkman A, Brug J. A systematic review of randomized trials on the effectiveness of computer-tailored education on physical activity and dietary behaviors. Annals of Behavioral Medicine 2006; 31(3):205-223.
(42)Webb T, Sheeran P. Does changing behavioral intentions engender behavior change? A meta-analysis of the experimental evidence. Psychological Bulletin 2006; 132:249-268.
(43)Orbell S, Hagger M, Brown V, Tidy J. Comparing two theories of health behavior: A prospective study of noncompletion of treatment following cervical cancer screening. Health Psychol 2006; 25 (5):604-615.
(44)Armitage C, Connor M. Armitage CJ, Conner M. Social cognition models and health behaviour: a structured review. Psychology & Health 2000; 15:173-189.
(45)Kok G, Schaalma H. Using theory in psychological interventions. In: Michie S, Abraham C, editors. Health psychology in practice. 1 ed. Oxford: Blackwell; 2004. 203-209.
(46)Milne S, Sheeran P, Orbell S. Prediction and intervention in health-related behavior: a meta-analytic review of protection motivation theory. Journal of Applied Social Psychology, 2000; 30:106-143.
(47)Rodgers R. Cognitive and physiological processes in fear appeals and attitude change: a revised

theory of protection motivation. In: Cacioppo J, Petty R, Shapiro D, editors. *Social Psychophysiology*. 1 ed. London: Guildford; 1983. 153-176.
(48)Witte K, Allen M. A meta-analysis of fear appeals: implications for effective public health campaigns. Health Education & Behavior 2006; 27:591-615.
(49)Tang C, Wong C. Factors influencing the wearing of facemasks to prevent the severe acute respiratory syndrome among adult Chinese in Hong Kong. Preventive Medicine 2004; 39:1187-1193.
(50)Leventhal H, Brissette I, Leventhal E. The common-sense model of self-regulation of health and illness. In: Cameron L, Leventhal H, editors. *The self-regulation of health and illness behaviour*. 1 ed. London: Routledge; 2003. 42-65.
(51)Slaughter L, Keselman A, Kushniruk A, Patel V. A framework for capturing the interactions between laypersons' understanding of disease, information gathering behaviors, and actions taken during an epidemic. Journal of Biomedical Informatics 2005; 38:298-313.
(52)Sheeran P, Abraham C, Orbell S. Psychosocial Correlates of heterosexual Condom Use: a Meta-Analysis. Psych Bull 1999; 125:90-132.
(53)Conner M, Armitage C. Extending the Theory of Planned Behavior: A Review and Avenues for Further Research. Journal of Applied Social Psychology 1998; 28:1429.
(54)Michie S, Johnston M, Cockcroft A, Ellinghouse C, Gooch C. Uptake and impact of health screening for hospital staff. J Organisational Behav 1994.
(55)Cockcroft A, Gooch C, Ellinghouse C, Johnston M, Michie S. Evaluation of a programme of health measurements and advice among hospital staff. J Occup Health 1994.
(56) Bagozzi R, Edwards E. Goal setting and goal pursuit in the regulation of body weight. Psychology & Health 1998; 593-621:593-621.
(57)Hobbis I, Sutton S. Are techniques used in cognitive behaviour therapy applicable to behaviour change interventions based on the theory of planned behaviour? Journal of Health Psychology 2005; 10(1):7-18.
(58)Ziegelman J, Lippke S, Schwarzer R. Adoption and maintenance of physical activity: Planning interventions in young, middle-aged, and older adults. Psychology & Health 2006; 21(2):145-163.
(59)Prestwich A, Conner M, Lawton R, Bailey W, Litman J, Molyneaux V. Individual and collaborative implementation intentions and the promotion of breast self-examination. Psychology & Health 2006 2006; 21:143.
(60) Yardley L, Nyman S. Internet provision of tailored advice on falls prevention activities for older people: a randomized controlled evaluation. Health Promotion International (minor revisions requested: in press) 2006.
(61)Kraft P, Rise J, Sutton S, Roysamb E. Perceived difficulty in the theory of planned behaviour: Perceived behavioural control or affective attitude? British Journal of Social Psychology 2005; 44:479-496.
(62) Howie J, Heaney D, Maxwell M, Walker J, Freeman G, Rai H. Quality at general practice consultations: cross sectional survey. BMJ 1999; 319:738-743.
(63) Oenema A, Brug J, Lechner L. Web-based tailored nutrition education: Results of a randomized controlled trial. Health Education Research 2001; 16:647-660.
(64) Little PS, Gould C, Williamson.I., Warner G, Gantley M, Kinmonth AL. Reattendance and complications in a randomised trial of prescribing strategies for sore throat: the medicalising effect of prescribing antibiotics. B M J 1997; 315:350-352.
(65) Little P, et al. A pragmatic randomised controlled trial of two prescribing strategies for acute otitis media. BMJ 2001; 322:336-342.
(66)Little PS, Williamson I, Warner G, Gould C, Gantley M, Kinmonth AL. An open randomised trial of prescribing strategies for sore throat. B M J 1997; 314:722-727.
(67)Lee G, Salomon J, Friedman J, Hibberd P, Ross-Degnan D, Zasloff E et al. Illness Transmission in the Home: A Possible Role for Alcohol-Based Hand Gels. Pediatrics 2005; 115:852-860.
(68)Hay A, Fahey T, Peters T, Wilson A. Predicting complications from acute cough in pre-school children in primary care: a prospective cohort study. B J G P 2004; 54:9-14.
(69)Sutton S. Predicting and explaining intentions and behaviour: how well are we doing. Journal of Applied Social Psychology 1998; 15:1317-1338.

(70)Aguilera JFea. Heterogeneous case definitions used for the surveillance of influenza in Europe. European Journal of Epidemiology 2003; 18:733-736.
(71)Balicer R, Huerta M, Levy Y, Davidovitch N, Grotto I. Influenza Outbreak Control in Confined Settings. Emerging Infectious disease 2005; 11 (4):http://www.cdc.gov/ncidod/eid/vol11no04-04-0845.htm.
(72)Boivin Gea. Predicting Influenza Infections during Epidemics with Use of a Clinical Case Definition. Clinical Infectious Disease 2000; 31:1166-1169.
(73)Nichol K, Mendelman P. Influence of clinical case definitions with differing levels of sensitivity and specificity on estimates of the relative and absolute health benefits of influenza vaccination among healthy working adults and implications for economic analyses. Virus Research 2004; 103, Issues 1-2:3-8.
(74)Rogers M. A viable alternative to the glass/mercury thermometer. Paed Nurs 1992; 4(9).
(75) Centor RM, Witherspoon JM, Dalton HP. The diagnosis of strep throat in the emergency room. Med Decis Making 1981; 1:239-246.
(76)Dobbs F. A scoring system for predicting Group A streptococcal throat infection. B J G P 1996; 46:461-464.
(77) Breese B. A simple scorecard for the tentative diagnosis of streptococcal pharyngitis. Am J Dis Child 1977; 131:514-517.
(78)Kind P, Hardman G,Macran S. UK population norms for EQ-5D. Discussion paper 172. 1216. York: Centre for Health Economics, University of York; 1999.
(79)Acute viral infections of upper respiratory tract in elderly people living in the community: comparative, prospective, population based study of disease burden. BMJ 1997; 315:1060-1064.
(80)Szucs T. The socio-economic burden of influenza. JAC 44, Topic B 11-15. 1999.
(81)Hayden FG et al. Inhaled Zanamivir for the Prevention of Influenza in Families. N Engl J Med 2000; 343:1282-1289.
(82)Nicholson et al. Risk factors for lower respiratory complications of rhinovirus infections in elderly people living in the community. B M J 1996; 313:1119-1123.
(83)Little P et al. A RCT of leaflets to empower patients in consultations. BMJ 2004; 328:441-444.
(84) Shah S, Sander S, White C, Rinaldi M, Coleman C. Evaluation of Echinacea for the prevention and treatment of the common cold: a meta-analysis. The Lancet Infectious Diseases 2007; 7:473
(85) Douglas R, Chalker E, Treacy B. Vitamin C for preventing and treating the common cold (Cochrane Review). Cochrane Library 2004.
(86) Timmer A, Gunther J, Rucker G, Motschall E, Antes G, Kern W. Pelargonium sidoides extract for acute respiratory tract infections. Cochrane Database of systematic reviews 2008; Issue 3. Art. No.: CD006323. DOI: 10.1002/14651858.CD006323.pub2.
(87) Singh M. Heated, humidified air for the common cold. Cochrane Library 2004; ammendment April 1999
